# Anaerobic gut fungal communities in marsupial hosts

**DOI:** 10.1128/mbio.03370-23

**Published:** 2024-01-23

**Authors:** Adrienne L. Jones, Carrie J. Pratt, Casey H. Meili, Rochelle M. Soo, Philip Hugenholtz, Mostafa S. Elshahed, Noha H. Youssef

**Affiliations:** 1 Department of Microbiology and Molecular Genetics, Oklahoma State University, Stillwater, Oklahoma, USA; 2 School of Chemistry and Molecular Biosciences, Australian Centre for Ecogenomics, The University of Queensland, St Lucia, Queensland, Australia; University of Minnesota Medical School, Minneapolis, Minnesota, USA

**Keywords:** marsupials, anaerobic fungi, community structure

## Abstract

The anaerobic gut fungi (AGF) inhabit the alimentary tracts of herbivores. In contrast to placental mammals, information regarding the identity, diversity, and community structure of AGF in marsupials is extremely sparse. Here, we characterized AGF communities in 61 fecal samples from 10 marsupial species belonging to four families in the order Diprotodontia: Vombatidae (wombats), Phascolarctidae (koalas), Phalangeridae (possums), and Macropodidae (kangaroos, wallabies, and pademelons). An amplicon-based diversity survey using the D2 region of the large ribosomal subunit as a phylogenetic marker indicated that marsupial AGF communities were dominated by eight genera commonly encountered in placental herbivores (*Neocallimastix*, *Caecomyces*, *Cyllamyces*, *Anaeromyces*, *Orpinomyces*, *Piromyces*, *Pecoramyces*, and *Khoyollomyces*). Community structure analysis revealed a high level of stochasticity, and ordination approaches did not reveal a significant role for the animal host, gut type, dietary preferences, or lifestyle in structuring marsupial AGF communities. Marsupial foregut and hindgut communities displayed diversity and community structure patterns comparable to AGF communities typically encountered in placental foregut hosts while exhibiting a higher level of diversity and a distinct community structure compared to placental hindgut communities. Quantification of AGF load using quantitative PCR indicated a significantly smaller load in marsupial hosts compared to their placental counterparts. Isolation efforts were only successful from a single red kangaroo fecal sample and yielded a *Khoyollomyces ramosus* isolate closely related to strains previously isolated from placental hosts. Our results suggest that AGF communities in marsupials are in low abundance and show little signs of selection based on ecological and evolutionary factors.

**IMPORTANCE:**

The AGF are integral part of the microbiome of herbivores. They play a crucial role in breaking down plant biomass in hindgut and foregut fermenters. The majority of research has been conducted on the AGF community in placental mammalian hosts. However, it is important to note that many marsupial mammals are also herbivores and employ a hindgut or foregut fermentation strategy for breaking down plant biomass. So far, very little is known regarding the AGF diversity and community structure in marsupial mammals. To fill this knowledge gap, we conducted an amplicon-based diversity survey targeting AGF in 61 fecal samples from 10 marsupial species. We hypothesize that, given the distinct evolutionary history and alimentary tract architecture, novel and unique AGF communities would be encountered in marsupials. Our results indicate that marsupial AGF communities are highly stochastic, present in relatively low loads, and display community structure patterns comparable to AGF communities typically encountered in placental foregut hosts. Our results indicate that marsupial hosts harbor AGF communities; however, in contrast to the strong pattern of phylosymbiosis typically observed between AGF and placental herbivores, the identity and gut architecture appear to play a minor role in structuring AGF communities in marsupials.

## INTRODUCTION

Marsupials (infraclass Marsupialia) are mammals characterized by giving birth to undeveloped offspring and caring for them in pouches. Marsupials represent the only extant group of metatherian mammals and are endemic to Australia and North and South America. Extant marsupials include herbivores (order Diprotodontia), carnivores (order Dasyuromorphia), and omnivores (orders Didelphimorphia and Peramelemorphia). The majority of marsupial herbivores, with rare exceptions such as the woolly opossum (genus *Caluromys*), are native to Australia and belong to the order Diprotodontia. Marsupial herbivores display a wide range of dietary preferences including browsers (feeding on trees and shrubs of high-growing plants, some of which can display high preference for leaves, i.e., folivores, or fruits, i.e., frugivores), grazers (feeding on grass and low-growing vegetation), and mixed feeders (1–3) (Table 1).

**TABLE 1:** Marsupials sampled in this study, with a description of their families, species, gut type, nutritional type, and habitat

Animal	Family	Species	Gut type	Nutritional type	Habitat	Number of animals/habitat
Eastern gray kangaroo	Macropodidae	*Macropus giganteus*	Foregut	Grazer	Sanctuary, Australia	4
Red kangaroo		*Osphranter rufus*	Foregut	Grazer	Zoo, USA	2
					Sanctuary, Australia	7
Red-legged pademelon		*Thylogale stigmatica*	Foregut	Mixed feeder	Sanctuary, Australia	1
Red-necked wallaby		*Notamacropus rufogriseus*	Foregut	Mixed feeder	Zoo, USA	3
					Sanctuary, Australia	3
Common brushtail possum	Phalangeridae	*Trichosurus vulpecula*	Hindgut	Mixed feeder	Sanctuary, Australia	1
Koala	Phascolarctidae	*Phascolarctos cinereus*	Hindgut	Folivore	Sanctuary, Australia	30
					Zoo, Australia	1
Common wombat	Vombatidae	*Vombatus ursinus*	Hindgut	Grazer	Sanctuary, Australia	3
					Zoo, Australia	1
Southern hairy-nosed wombat		*Lasiorhinus latifrons*	Hindgut	Grazer	Sanctuary, Australia	4
					Zoo, Australia	1

Similar to placental mammals (infraclass Placentalia), marsupial herbivores rely on microorganisms in their gastrointestinal tract for plant digestion and conversion to absorbable fermentation end products (2, 4). In both groups, fermentation occurs in specialized chambers with extended food retention times to enable colonization, plant polymer mobilization and breakdown, and monomer/oligomer fermentation to soluble end products by the resident microbiota. However, herbivorous marsupial and placental guts are structurally distinct. Marsupial foregut fermenters (members of the family Macropodidae, e.g., kangaroos, wallabies, wallaroos, and pademelons) possess an enlarged forestomach region divided into an anterior sacciform and posterior tubiform, with fermentation processes occurring in both regions (4). In contrast, the majority of fermentation processes in placental foregut fermenters occurs in the rumen, a pregastric chamber that represents part of a complex four-chambered stomach. Hindgut marsupial fermenters have an enlargement of a variety of intestinal region(s), with some possessing an enlarged colon (e.g., wombats), caecum (e.g., possums), or both colon and caecum (e.g., koalas). Furthermore, marsupial herbivores in general have a relatively lower basal metabolic rate and display an ability to forage on poor nutritional diets compared to placental mammals; adaptations seen as necessary for survival in poorly productive and arid habitats and a highly variable climate (5).

Multiple studies have investigated microbial communities in various marsupials using culture-based (6–8), amplicon-based (9–11), and omics-based approaches (12–16). These studies have identified prevalent bacterial lineages in the gut of various herbivorous marsupial taxa and yielded valuable insights into the impact of ecological and evolutionary factors in shaping marsupial gut bacterial communities. However, while we have a baseline of knowledge concerning the bacterial and archaeal components of the marsupial gut, the prevalence, identity, and community structure of anaerobic gut fungi (AGF) are currently unclear.

The AGF belong to a distinct basal fungal phylum (Neocallimastigomycota) (17) and were discovered in the rumen of sheep in 1975 (18). They were subsequently shown to be key constituents of the gut microbiomes of a wide range of placental mammalian herbivores (19). As previously noted (19), establishment of AGF in the gut of a herbivorous host requires long retention times and a dedicated digestive chamber (e.g., rumen, forestomach, or caecum), criteria that are satisfied in herbivorous marsupials. However, our current knowledge regarding AGF communities in marsupial herbivores is extremely sparse. An earlier review alluded to unpublished efforts pertaining to the isolation of AGF from a red kangaroo (*Macropus rufus*) (20). The isolates were putatively identified as *Piromyces* species based on microscopic observations, although diagnostic features of the genus (monocentric thalli, filamentous hyphae, and monoflagellated zoospores) have since been observed in 13 additional genera (21–24). Another review also reported on unpublished efforts where AGF rhizoidal growth was observed on plant fragments from the stomachs of four macropod species: gray kangaroo (*Macropus giganticus*), red-necked wallaby (*Macropus rufogriseus*), wallaroo (*Macropus robustus*), and swamp wallaby (*Wallabia bicolor*) (25). In addition, two previous culture-independent amplicon surveys examined AGF communities in zoo-housed red kangaroo and white-fronted wallaby (*Osphranter rufus* and *Macropus parma*) and reported a diverse community affiliated with the genera *Piromyces*, *Anaeromyces*, *Khoyollomyces*, as well as multiple yet uncultured genera representing the most abundant AGF genera (26).

Here we sought to characterize AGF in marsupial herbivores (order Diprotodontia) using culture-independent diversity surveys, quantitative PCR (qPCR) quantification, and enrichment and isolation procedures. We hypothesized that the distinct gut architecture and dietary preferences of marsupial herbivores, as well as their unique evolutionary history and geographic range restriction, could select for an AGF community characterized by a high proportion of novel taxa or distinct community structure patterns compared to those of placental mammals. Surprisingly, our results suggest that the AGF communities in marsupials are neither novel nor unique. Rather, AGF appear to be present in relatively small loads or absent in marsupial gut, in contrast to their ubiquity and higher loads in placental herbivores. Furthermore, AGF communities in marsupials appear to exhibit diversity and community structure patterns similar to those encountered in placental foregut fermenters. The ecological and evolutionary factors underpinning such observed patterns are discussed.

## MATERIALS AND METHODS

### Samples

Marsupial fecal samples included representatives of 6 different families (Macropodidae, Phascolarctidae, Phalangeridae, Petauridae, Pseudocheiridae, and Vombatidae), 15 different genera, and 20 different species in the order Diprotodontia (Table S1). These hosts encompass multiple different gut types (foregut fermenters, hindgut fermenters with an enlarged colon, caecum, or both), dietary classifications (browsers, grazers, and mixed feeders), and lifestyles (zoo housed and sanctuary housed). Of the 184 marsupial samples examined, only 61 yielded AGF amplicons despite repeated attempts (Table S1). Individual samples originated from a single animal and were not adulterated during sampling with dust, dirt, or feces from other subjects.

To compare marsupial AGF communities generated in this study to AGF communities from placental mammals, a data set of placental mammals comprising 25 cattle, 25 goats, 25 sheep, 20 horses, 7 elephants, 3 rhinoceroses, and 3 zebras was used (Table S2). These samples represent a fraction of samples included in a recent study of the placental AGF mycobiome (27). The data set was combined with the 61 marsupial samples reported here, and the mixed data set was analyzed for AGF alpha diversity and community structure as described below.

### DNA extraction and amplification

DNA extraction was conducted using a DNeasy Plant Pro kit (Qiagen, Germantown, MD, USA) according to manufacturer’s instructions. The kit has previously been evaluated and utilized by multiple laboratories in prior AGF diversity surveys (27–29). Amplification of the D2 region of the large ribosomal subunit (D2 LSU) was achieved using primer pair AGF-LSU-EnvS primer pair (AGF-LSU-EnvS for: 5′-GCGTTTRRCACCASTGTTGTT-3′, AGF-LSU-EnvS rev: 5′-GTCAACATCCTAAGYGTAGGTA-3′) (27, 29) modified to include the Illumina overhang adaptors. The large ribosomal subunit has been shown to be superior to ITS1, commonly used for diversity surveys of other fungal lineages, as it exhibits a much lower level of length and sequence divergence heterogeneity (28, 30) and is currently the standard phylomarker in diversity surveys of AGF (27–29). PCR reactions contained 2 µL of DNA, 25 µL of the DreamTaq 2× Master Mix (Life Technologies, Carlsbad, CA, USA), and 2 µL of each primer (10 µM) in a 50-µL reaction mix. The PCR protocol consisted of an initial denaturation for 5 min at 95°C followed by 40 cycles of denaturation at 95°C for 1 min, annealing at 55°C for 1 min and elongation at 72°C for 1 min, and a final extension of 72°C for 10 min. For samples showing negative PCR amplification in initial attempts, additional efforts were conducted (varying the DNA concentrations), and samples were only deemed negative after four attempts. Negative (reagents only) controls were included with all PCR amplifications to detect possible cross-contamination.

### Sequencing and sequence processing

PCR products were individually cleaned using PureLink gel extraction kit (Life Technologies) and indexed using Nextera XT index kit v2 (Illumina Inc., San Diego, CA, USA). Libraries were pooled using the Illumina library pooling calculator (https://support.illumina.com/help/pooling-calculator/pooling-calculator.htm), and pooled libraries were sequenced at the University of Oklahoma Clinical Genomics Facility (Oklahoma City, OK, USA) using the MiSeq platform and the 300-bp PE reagent kit. Forward and reverse Illumina reads were assembled using the make.contigs command in mothur (31), followed by removing sequences with ambiguous bases, homopolymer stretches longer than eight bases, and sequences that were shorter than 200 bp or longer than 380 bp. A two-tier approach, as detailed before (27, 28), was used to confidently assign sequences to previously described genera and candidate genera or to novel candidate genera. These genus-level assignments were used to build a shared file using the mothur commands phylotype and make.shared, and the shared file was subsequently utilized as an input for downstream analysis.

### Alpha-diversity measures

Alpha-diversity estimates (Shannon, Simpson, and Inverse Simpson diversity indices) were calculated using the command estimate_richness in the phyloseq R package (32). To evaluate the importance of various factors in shaping alpha-diversity patterns, only samples with at least four replicates of the factors listed below were included. Comparisons were conducted between Macropodidae, Phascolarctidae, and Vombatidae (for host family comparison); red kangaroo, eastern gray kangaroo, koala, red-necked wallaby, southern hairy-nosed wombat, and common wombat (for the animal species comparison); foregut and hindgut (for the gut type factor comparison); sanctuary and zoo (for habitat comparison); and grazer, foliovore, and mixed-feeder (for nutritional preferences comparisons). Nonparametric analysis of variance (ANOVA) (calculated using the kruskal.test command in R) followed by post hoc Dunn tests [when significant, using dunnTest command in the FSA R package (33)] were used for multiple comparisons of means to identify the pairs of groups that are significantly different for each host factor. In addition, the same alpha-diversity estimates were compared to AGF alpha-diversity patterns in a subset of placental counterparts [25 cattle, 25 goats, 25 sheep, 20 horses, 7 elephants, 3 rhinoceroses, and 3 zebras was used (Table S2)]. These placental samples represent a fraction of samples included in a recent study of the placental AGF mycobiome (27). Nonparametric ANOVA (calculated using the kruskal.test command in R) followed by post hoc Dunn tests (when significant, using dunnTest command in the FSA R package) were used to identify the the pairs of gut type/infraclass combinations that are significantly different (foregut marsupial versus foregut placental, foregut marsupial versus hindgut placental, hindgut marsupial versus foregut placental, and hindgut marsupial versus hindgut placental).

### Community assembly and stochasticity

Assembly and structuring of microbial communities could be governed by deterministic (niche theory-based) or stochastic (null theory-based) processes (34–36). Two approaches were utilized to examine the contribution of various deterministic and stochastic processes in shaping community assembly: the normalized stochasticity ratio (NST) (34), and the null-model-based quantitative framework [implemented by references (35, 36)]. The normalized stochasticity ratio was calculated using the NST package in R (34) based on two taxonomic beta-diversity dissimilarity metrics: the incidence-based Jaccard index and the abundance-based Bray-Curtis index. The function nst.boot in the NST package in R was then used to randomly draw samples within each comparison group, followed by bootstrapping of NST values. The values obtained after bootstrapping were then compared using Wilcoxon test with Benjamini-Hochberg adjustment. The iCAMP R package (37) was used to calculate values of beta net relatedness index (βNRI) and modified Raup-Crick metric based on Bray-Curtis metric (RC_Bray_) using the function bNRIn.p. Values of βNRI and RC_Bray_ were used to partition selective processes into homogenous and heterogenous selection and stochastic processes into dispersal and drift as detailed before (27). The percentages of pairwise comparisons falling into each category were used as a proxy for the contribution of each of these processes (homogenous selection, heterogenous selection, homogenizing dispersal, and drift) to the total AGF community assembly.

### Community structure

The phylogenetic similarity-based weighted Unifrac index was calculated using the ordinate command in the phyloseq R package and the pairwise values were used to construct principal coordinate analysis (PCoA) ordination plots using the function plot_ordination in the phyloseq R package. Permutational multivariate analysis of variance (PERMANOVA) tests were run using the command adonis in the vegan R package (38). Host factors (family, species, gut type, and nutritional preference), as well as habitat, were tested individually (with no interaction terms). The *F*-s*t*atistics *P* values were compared to identify factors that significantly affect the AGF community structure. The percentage variance explained by each factor was calculated as the percentage of the sum of squares of each factor to the total sum of squares.

The AGF community structure in marsupial hosts was also compared to the placental AGF community structure in the same placental data set used for alpha-diversity comparisons (see above). PERMANOVA tests (run using the command adonis in the vegan R package) were used to partition the dissimilarity among the host infraclass (Marsupialia versus Placentalia) and gut type (foregut versus hindgut), with the addition of interaction terms (to test for gut type-specific differences in the host infraclass).

### Quantitative PCR

AGF loads were quantified in 43 samples (10 kangaroos, 18 koalas, 5 wallabies, 9 wombats, and 1 pademelon) (samples color coded in red in Table S1) using qPCR targeting the D2 region of the LSU rRNA (29). The 25-µL PCR reaction volume contained 2 µL of extracted DNA, 0.3 µM of primers AGF-LSU-EnvS primer pair (AGF-LSU-EnvS for: 5′-GCGTTTRRCACCASTGTTGTT-3′ and AGF-LSU-EnvS rev: 5′-GTCAACATCCTAAGYGTAGGTA-3′) (29), and SYBR GreenER qPCR SuperMix for iCycler (Thermo Fisher, Waltham, MA, USA), and were run on a MyiQ thermocycler (Bio-Rad Laboratories, Hercules, CA, USA). The amplification protocol was composed of heating at 95°C for 8.5 min, followed by 40 cycles, with one cycle consisting of 15 s at 95°C and 1 min at 55°C. AGF were quantified in fecal samples as the number of LSU rRNA copies/g sample. The number of copies was calculated from the standard curve obtained from running pCR 4-TOPO or pCR-XL-2-TOPO plasmid (Thermo Fisher) containing an insert spanning ITS1-5.8S rRNA-ITS2-D1/D2 region of 28S rRNA from a pure culture strain.

In addition to marsupial samples, AGF loads were also quantified in the feces of 40 placental mammalian AGF hosts (10 cattle, 10 goats, 10 sheep, and 10 horses) for comparative purposes. Wilcoxon *t*-test (calculated using wilcox_test in the R package stats) was used to test the significance of difference between marsupial versus placental AGF loads, while nonparametric ANOVA (calculated using the kruskal.test command in R) was used to test the significance of difference of AGF load between different marsupial families, gut types, and species.

### Isolation of AGF from the marsupial gut

Isolation procedures were conducted as previously described (24). Isolation efforts were conducted at 35°C and 39°C using switchgrass, cellulose, or both as a substrate. In total, 40 different enrichments were set up using 18 different marsupial fecal samples. Isolation attempts were undertaken for enrichments showing positive growth using the roll tube method as described earlier (39). Isolates from the one successful enrichment were maintained at 39°C and identified using PCR and sequencing of the D1/D2 LSU using NL1 and NL4 primers as previously described (40). To assess the phylogenetic position of the newly obtained isolates, we used D1/D2 LSU as phylogenetic markers. Sequences were aligned to reference AGF sequences using mafft (41), and the alignment was used to construct a maximum likelihood phylogenetic tree in FastTree (42).

### Sequence and data deposition

Illumina reads were deposited in GenBank under BioProject accession number PRJNA978249. Sequences of the D1/D2 region of the 28S rRNA from the five isolates were submitted to GenBank under accession numbers OR072728–OR075732. All codes used to create figures and calculate statistics are available at https://github.com/nohayoussef/AGF_MArsupials.

## RESULTS

### Amplicon-based diversity survey overview

PCR amplification was successful from all or some samples belonging to eight different marsupial species and genera: red kangaroo (*Osphranter rufus*), eastern gray kangaroo (*Macropus giganteus*), koala (*Phascolarctos cinereus* subspecies *adustus*), red-legged pademelon (*Thylogale stigmatica*), common brushtail possum (*Trichosurus vulpecula*), red-necked wallaby (*Notamacropus rufogriseus*), southern hairy-nosed wombat (*Lasiorhinus latifrons*), and common wombat (*Vombatus ursinus*) (Table S1). AGF amplification failed from all samples belonging to 11 marsupial species: northern-tail wallaby (*Onychogalea unguifera*, *n* = 4), agile wallaby (*Notamacropus agilis*, *n* = 3), Bennet’s wallaby (*Macropus rufogriseus*, *n* = 2), swamp wallaby (*Wallabia bicolor*, *n* = 2), tammar wallaby (*Notamacropus eugenii*, *n* = 5), parma wallaby (*Notamacropus parma*, *n* = 1), common ringtail possum (*Pseudocheirus peregrinus*, *n* = 3), short-eared possum (*Trichosurus caninus*, *n* = 3), Lumholtz’s tree kangaroo (*Dendrolagus lumholtzi*, *n* = 4), squirrel glider (*Petaurus norfolcensis*, *n* = 1), and greater glider (*Petauroides armillatus*, *n* = 1) (Table S1).

### Community overview

**Fig 1:**
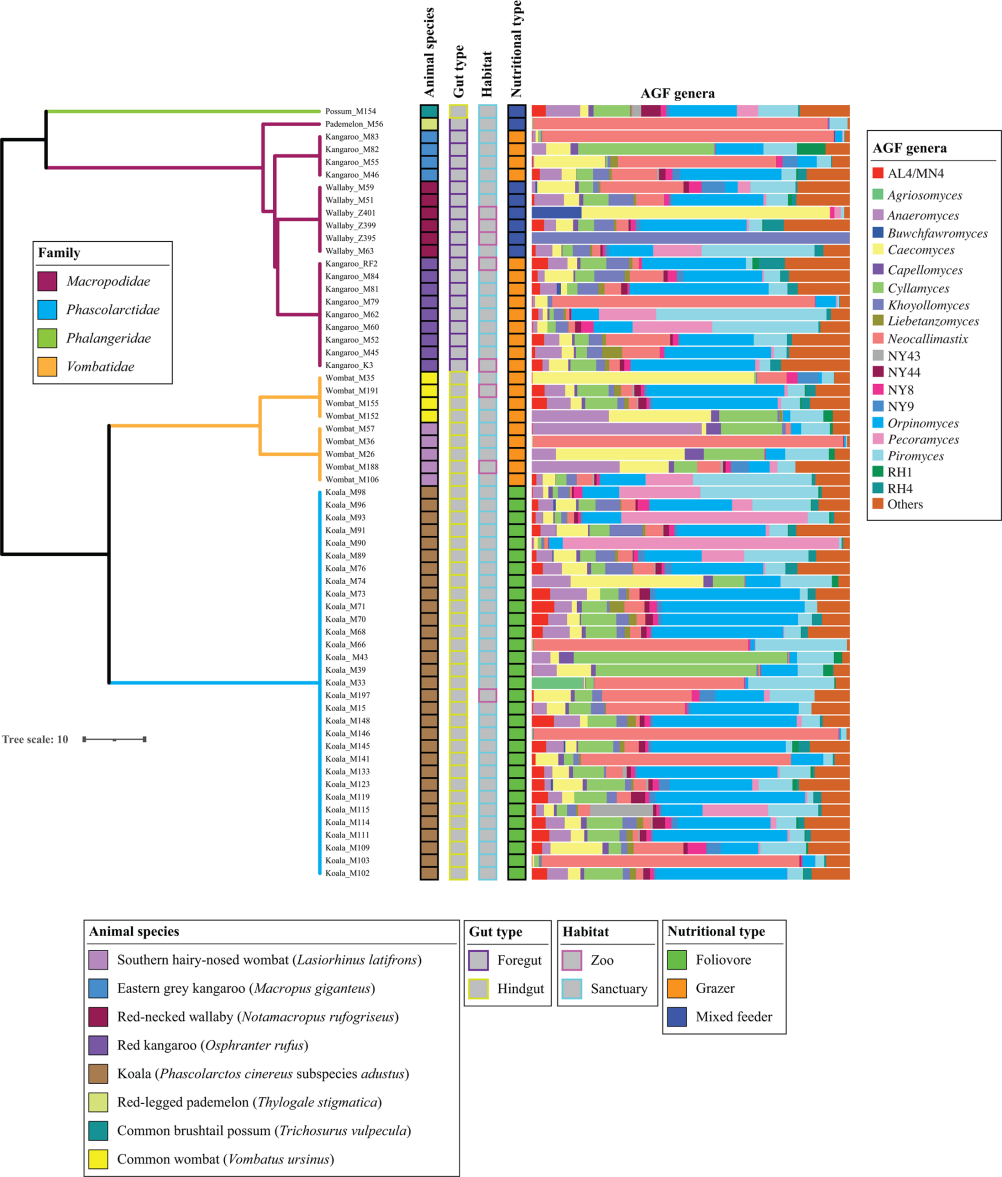
AGF community composition in the samples studied. The phylogenetic tree showing the relationship between animals was downloaded from timetree.org and modified to include very short branch length between samples from the same animal species. Branches are color coded by family as shown in the figure legend. Tracks to the right of the tree depict the species, gut type, habitat, and nutritional type of the animals studied as shown in the figure legend. AGF genera percentage of abundances is shown to the right of the tracks, with genera with <1% relative abundance grouped in “others.”

**Fig 2:**
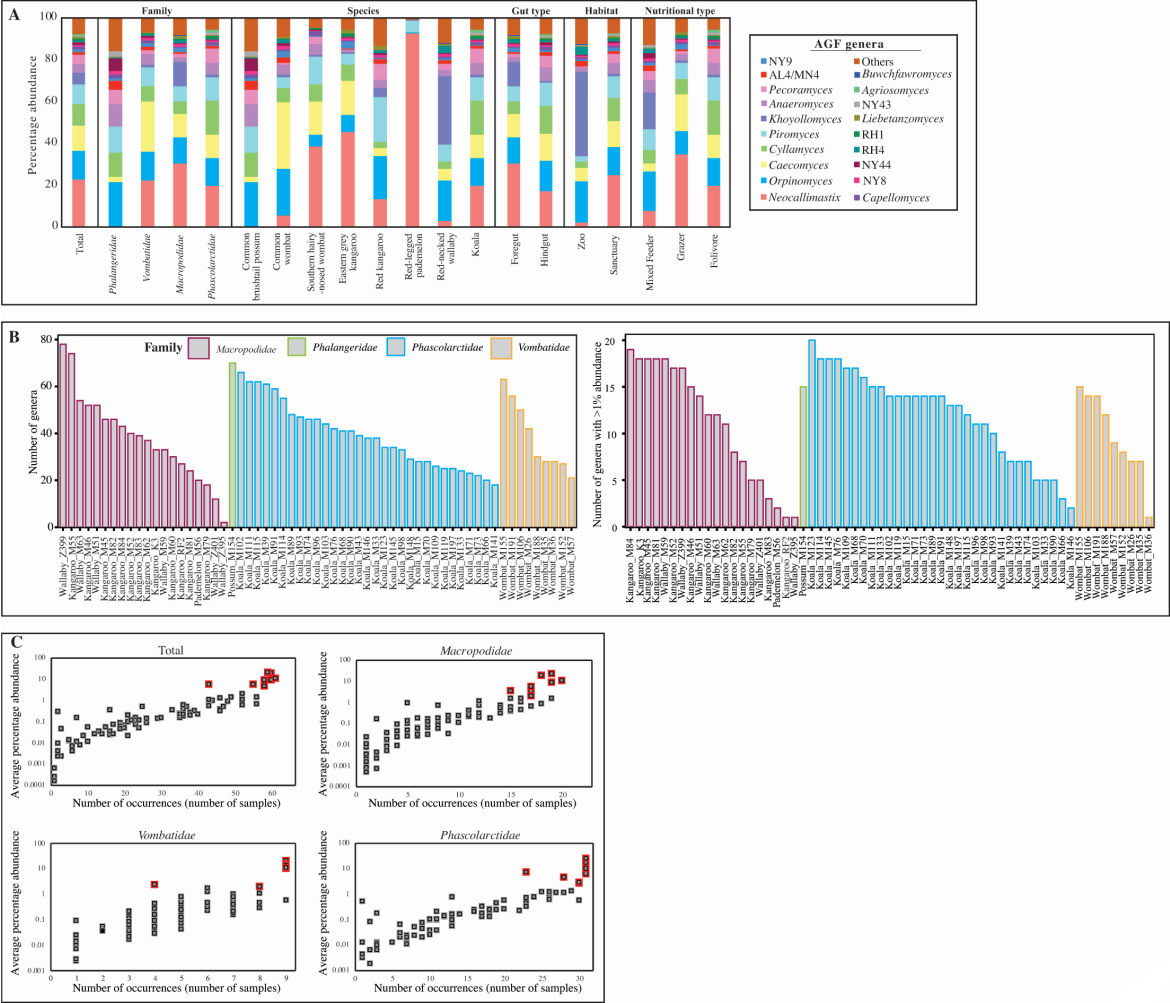
(**A**) AGF percentage of abundance shown for all samples studied, including families, species, gut type, habitat, and nutritional type. AGF genera with <1% relative abundance are grouped in “others.” (**B**) Total number of AGF genera (left) and number of AGF genera with >1% relative abundance (right) identified per sample. Samples names are shown on the *X*-axis (names match those in Fig. 1), and samples are grouped by the animal family as depicted in the figure legend. (**C**) Relationship between occurrence (number of samples) and average relative abundance of each of the 85 genera encountered in this study. The number of samples in which the genera was identified is shown on the *X*-axis. Average percentage of abundance across samples is plotted on the Y axis in a logarithmic scale to show genera present below 1% abundance. The eight mostly abundant genera (*Neocallimastix*, *Orpinomyces*, *Caecomyces*, *Cyllamyces*, *Piromyces*, *Khoyollomyces*, *Anaeromyces*, and *Pecoramyces*) are shown with a red border. Abundance-occurrence plots are shown for all samples studied, as well as for each of the three families with >5 animals, as depicted above each figure.

A total of 174,959 Illumina sequences of the D2 LSU region were obtained (average 2,868 ± 4,397 per sample; Table S3). Phylogenetic analysis of the entire data set demonstrated that, collectively, marsupials harbor a phylogenetically diverse AGF community. Representatives of 85 of the 87 currently reported AGF genera and candidate genera (27) were encountered (Fig. 1 and 2; Table S4). Only one additional novel genus (NY57) was identified as a ubiquitous (40 out of 61 samples), albeit minor (relative abundance 0.03%–1.27%, Table S4), component of the AGF community in marsupials. Within individual samples, a diverse, multigenus AGF community was observed, with an average number of genera ranging between 12 and 78 (1–20 if only considering genera present in >1% relative abundance) (Fig. 2B).

Phylogenetically, eight AGF genera represented the majority (79.33%) of the marsupial AGF communities in the entire data set: *Orpinomyces* (19.66% ± 16.1%), *Neocallimastix* (17.23% ± 28.4%), *Piromyces* (10.04% ± 10.9%), *Caecomyces* (8.75% ± 14.77%), *Cyllamyces* (8.18% ± 11.66%), *Anaeromyces* (5.47% ± 8.05%), *Pecoramyces* (5.24% ± 14.2%), and *Khoyollomyces* (4.23% ± 12.72%) (Fig. 2A). The predominance of these genera was observed across the marsupial families Macropodidae (75.55%), Phascolarctidae (85.6%), and Vombatidae (84.65%). In addition to their high relative abundance, these eight genera were also ubiquitous across the three families (Fig. 2C, red boxes), with a positive correlation observed between relative abundance and prevalence.

### Alpha-diversity estimates

**Fig 3:**
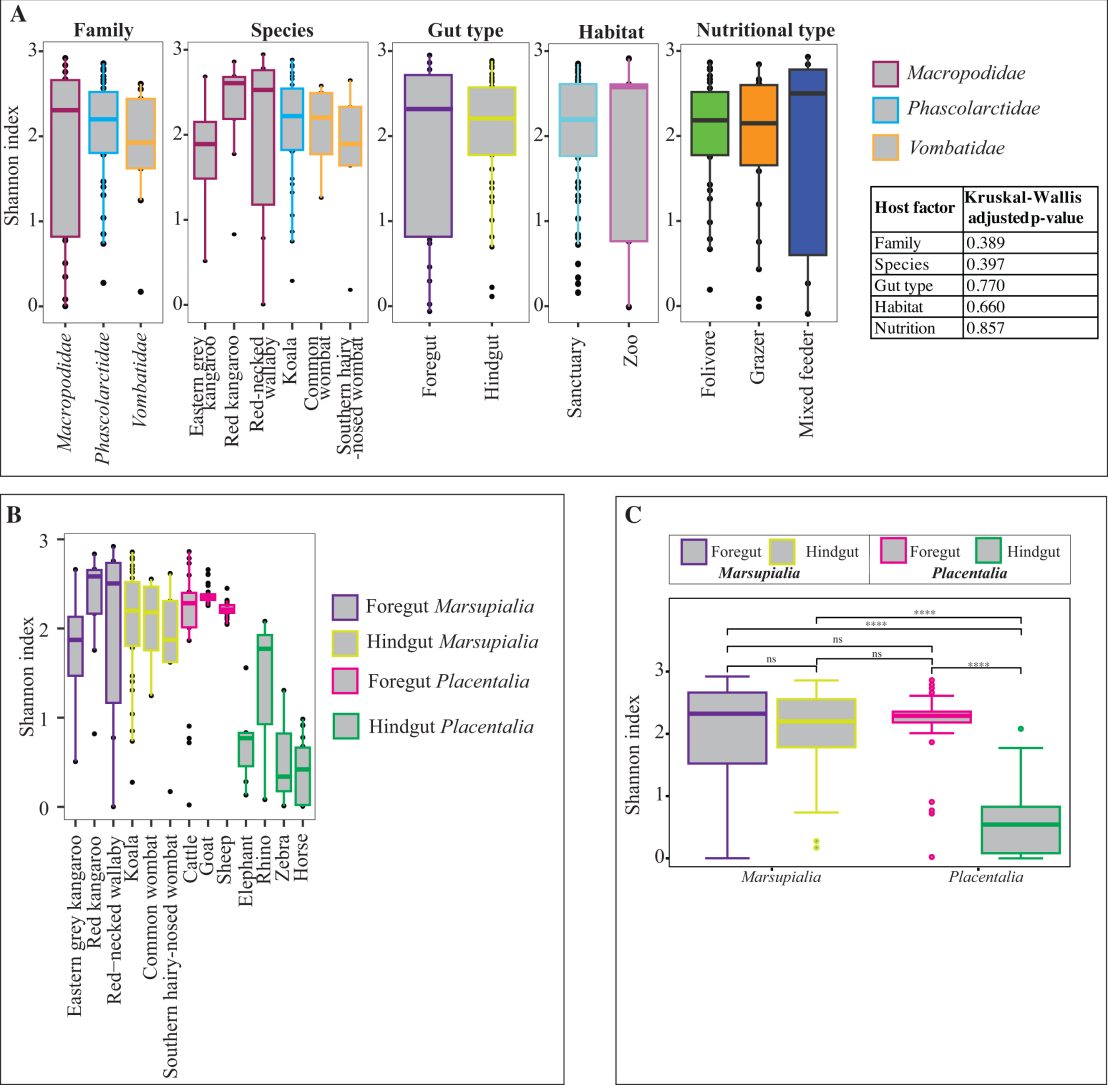
Alpha-diversity patterns. (**A**) Box and whisker plots showing the distribution of Shannon diversity index for different families, species, gut types, habitats, and nutritional types of the animals studied. Results of the Kruskal-Wallis test are shown in the table to the right. (**B**) Box and whisker plots showing the distribution of Shannon diversity index for animals species, color coded by their gut type, in comparison with foregut and hindgut placental animal representatives. (**C**) Results of Dunn post hoc tests for pairwise infraclass gut-type comparisons. ****P < 0.0001. ns, not significant.

AGF alpha-diversity patterns were assessed using three different indices: Shannon (Fig. 3), Simpson, and inverse Simpson (Fig. S1). Nonparametric ANOVA (Kruskal-Wallis) results showed a comparable level of alpha-diversity between all families and species examined, as well as between foregut and hindgut fermenters, zoo- and sanctuary-housed animals, and nutritional types (Fig. 3A; Fig. S1a; Table S5).

In addition to comparing alpha-diversity patterns among various marsupial hosts, we also compared marsupial AGF alpha-diversity patterns to their placental counterparts. The results indicated that all marsupial species (regardless of their gut type) harbor an AGF community with a comparable alpha diversity (Kruskal-Wallis followed by Dunn post hoc test, *P* value = 1) to placental foregut fermenters (Fig. 3B; Fig. S1b and c; Table S6) and a significantly higher alpha-diversity than placental hindgut fermenters (Kruskal-Wallis followed by Dunn post hoc test, *P* value < 3.2×10^−7^) (Fig. 3B; Fig. S1b and c; Table S7).

### Stochastic processes play an important role in shaping AGF community in marsupials

**Fig 4:**
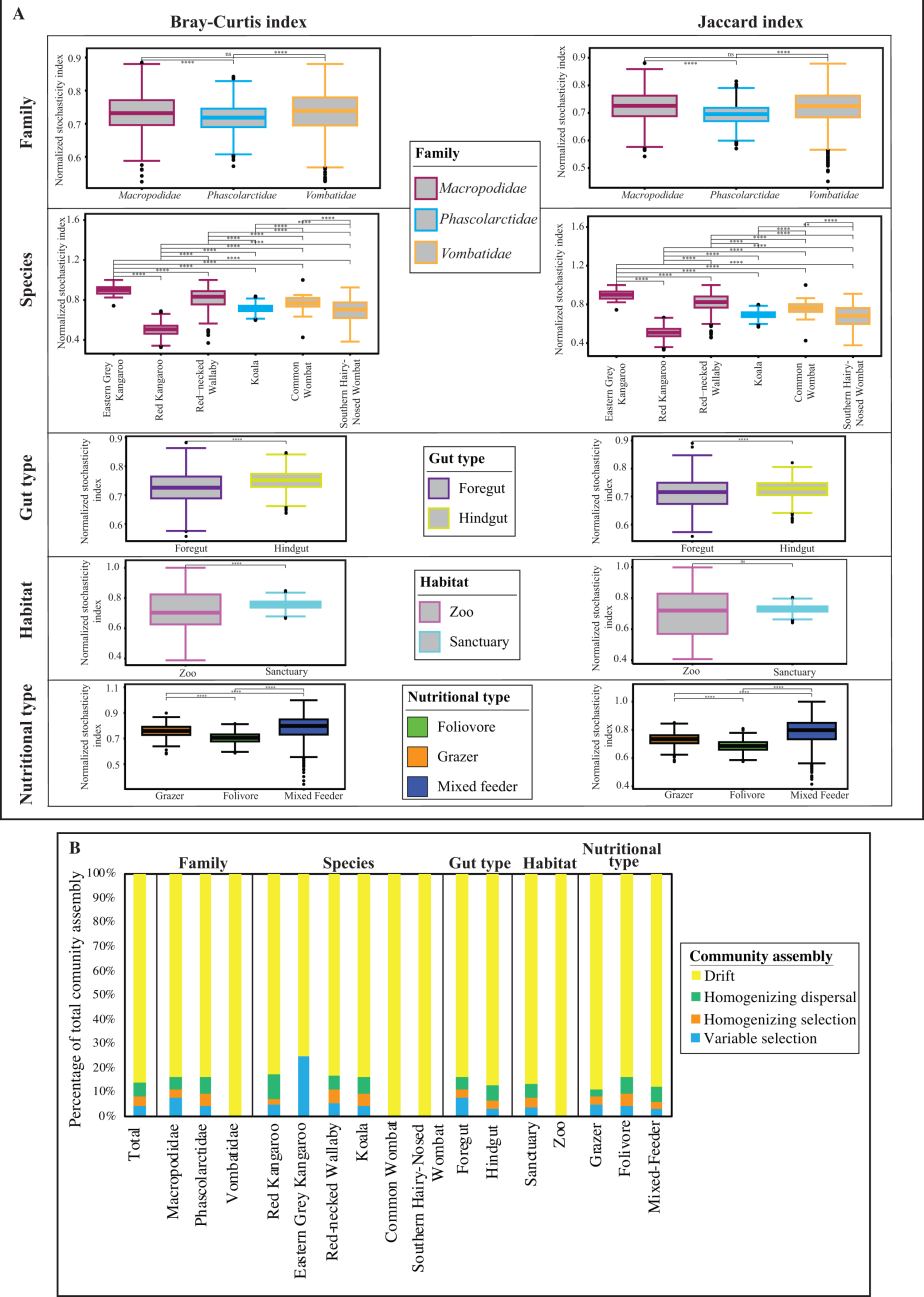
AGF community assembly in marsupial hosts. (**A**) Box and whisker plots showing the distribution of the bootstrapping results (*n* = 1,000) for the levels of stochasticity in AGF community assembly calculated as NST. Results compare different animal families (top row), animal species (second row), gut type (third row), habitat (fourth row), and nutritional type (fifth row). Two NSTs were calculated: the abundance-based Bray-Curtis index (left) and the incidence-based Jaccard index (right). Wilcoxon test, P value: **, 0.001< p < 0.01; ****, p < 0.0001. Details about how these results were obtained are explained in Materials and Methods. (**B**) The percentages of the various deterministic and stochastic processes shaping AGF community assembly of the total data set, and when subsetting for different animal families, species, gut types, habitats, and nutritional types. ns, not significant; NST, normalized stochasticity ratio.

NSTs indicate that, regardless of the β-diversity index used (abundance-based Bray-Curtis index, and incidence-based Jaccard index), stochastic, rather than deterministic, processes are the major contributors to AGF community assembly in marsupials (NST values of 70-75.7% for families, 51.4-90% for species, 72.5-76% for gut type, 69.9-76.4% for habitat, and 69.4-78.5% for nutritional type) (Fig. 4A). AGF community assembly in the different marsupial species significantly differed in their stochasticity, with values increasing in the order: red kangaroo <southern hairy-nosed wombat <koala < common wombat <red-necked wallaby <eastern gray kangaroo (Wilcoxon test *P*-value < 0.05) (Fig. 4A; Table S8).

To quantify the contribution of specific stochastic (homogenizing dispersal, dispersal limitation, and drift) processes in shaping the AGF community assembly in marsupials, we employed the previously suggested two-step null-model-based quantitative framework (35, 36). Results (Fig. 4B) broadly confirmed the patterns observed with NST values, where the AGF community assembly is mostly stochastic. The majority of stochasticity is caused by drift across all host species, families, gut types, habitats, and nutritional types examined (Fig. 4B).

### Community structure patterns

**Fig 5:**
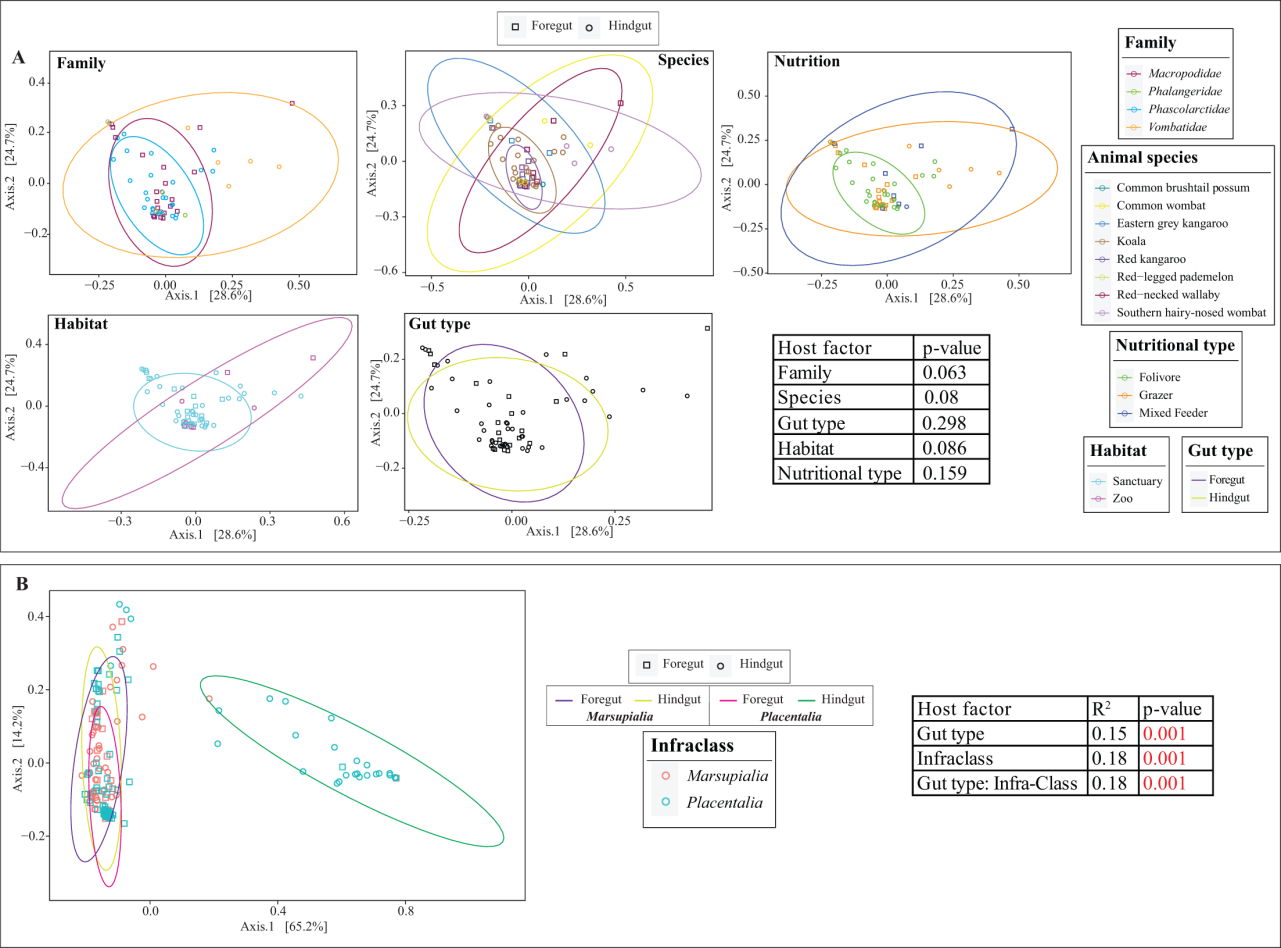
Ordination plots based on AGF community structure in the studied hosts. (**A**) Principal coordinate analysis ordination plots based on AGF community structure were constructed using the phylogenetic similarity-based weighted Unifrac index. Samples are color coded by animal family, species, nutritional type, habitat, and gut type as shown in the legend on the right-hand side, while the shape depicts the gut type as shown in the figure legend on top. Ellipses encompassing 95% of variance are shown for each of the factors and are color coded similar to the samples. Results of PERMANOVA test for partitioning the dissimilarity among the sources of variation are shown in the table to the right. The *F-*statistic *R*^2^ depicts the fraction of variance explained by each factor, while the *P* value depicts the significance of the host factor in affecting the community structure. (**B**) AGF community structure in marsupial hosts in comparison to placental mammals. Variance is shown for the four subcategories (foregut Marsupialia, foregut Placentalia, hindgut Marsupialia, and hindgut Placentalia). Results of PERMANOVA test for partitioning the dissimilarity are shown in the table to the right.

AGF community structure in marsupials was assessed using PCoA based on the phylogenetic similarity-based beta-diversity index weighted Unifrac. The first two axes explained 53.3% of the variance. The results demonstrated no clear role for marsupial family (Fig. 5A; PERMANOVA *F*-statistic = 1.731, df = 3, sum of squares = 0.326, *P* value = 0.053); species (Fig. 5A, PERMANOVA *F*-statistic = 1.577, df = 7, sum of squares = 0.673, *P* value = 0.08); gut type (Fig. 5A, PERMANOVA *F*-statistic = 1.15, df = 1, sum of squares = 0.075, *P* value = 0.33); habitat (Fig. 5A, PERMANOVA *F*-statistic = 2.201, df = 2, sum of squares = 0.275, *P* value = 0.093); or nutritional type (Fig. 5A, PERMANOVA *F*-statistic = 1.41, df = 2, sum of squares = 0.181, *P* value = 0.167) in shaping AGF community structure in marsupials.

However, when marsupials’ AGF community structure was compared to that of placental mammals, all marsupial samples showed a clear clustering pattern close to foregut placental hosts, with hindgut placental host clustering separately (Fig. 5B). To partition the dissimilarity among the sources of variation (host infraclass and gut type), we ran PERMANOVA tests (43) with the addition of interaction terms (to test for gut type specific differences in the host infraclass). Host infraclass, gut type, and the interaction of both, all significantly influenced community structure (*F*-statistics = 59.39, 50.79, 59.17, respectively; df = 1; sum of squares = 4.38, 3.74, and 4.36, respectively; *P* value = 0.001), with the largest effect being the infraclass (18% of variance), and its interaction with gut type (18% of variance).

### AGF loads in marsupial hosts

**Fig 6:**
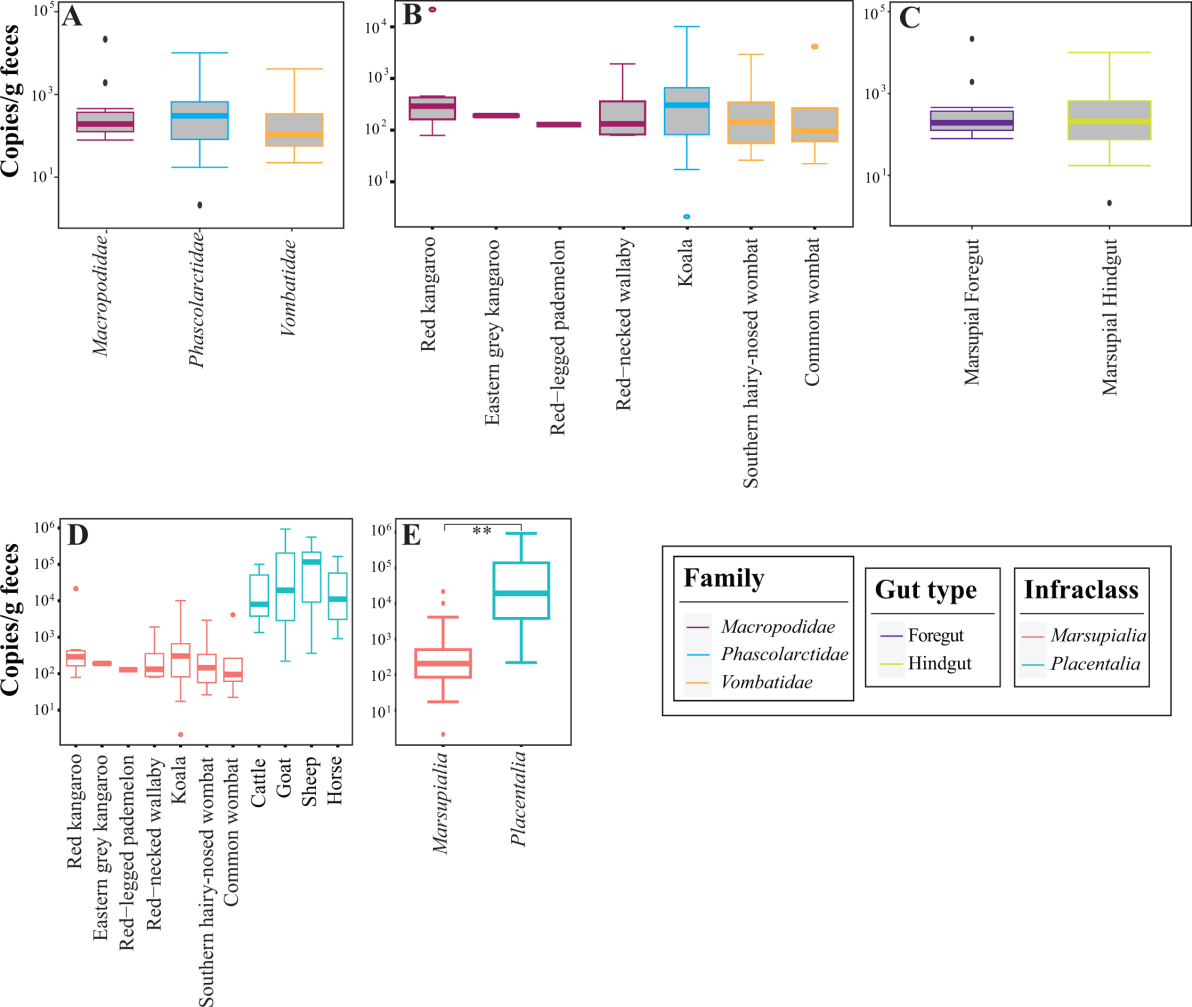
AGF load in the 43 marsupial samples examined using quantitative PCR. Boxplots showing the distribution of AGF load in the three marsupial families (**A**) seven marsupial species (**B**) and two gut types (**C**). (**D**) Comparison to AGF load in the 43 marsupial hosts to 40 placental counterparts. Boxplots in panel **E** show the distribution of AGF load in the 43 marsupial hosts (infraclass Marsupialia) versus the 40 placental hosts (infraclass Placentalia). **Wilcoxon *t*-test, *P* value = 0.0012.

AGF load was tested in 43 samples representing the 3 well-sampled marsupial families Macropodidae, Phascolarctidae, and Vombatidae, as well as 7 of the 8 marsupial species studied here [red kangaroo (*n* = 8), eastern gray kangaroo (*n* = 2), red-legged pademelon (*n* = 1), red-necked wallaby (*n* = 5), koala (*n* = 18), common wombat (*n* = 4), and southern hairy-nosed wombat (*n* = 5)]. AGF load in all examined marsupials was low (1.19 × 10^2^ ± 3.6 × 10^2^ copies/g feces). No significant differences were observed based on animal family (Kruskal-Wallis test, *P* value = 0.67), species (Kruskal-Wallis test, *P* value = 0.86), or gut type (Kruskal-Wallis test, *P* value = 0.67) (Fig. 6A through C; Table S9).

For comparison, AGF load quantified in 40 placental samples (representing 10 cattle, 10 goat, 10 sheep, and 10 horses) was significantly higher (average = 1.01 × 10^5^ ± 1.82 × 10^5^ copies/g feces) compared to marsupial mammals (Wilcoxon test, *P* value = 0.0012) (Fig. 6D and E; Table S10).

### Isolation

**Fig 7:**
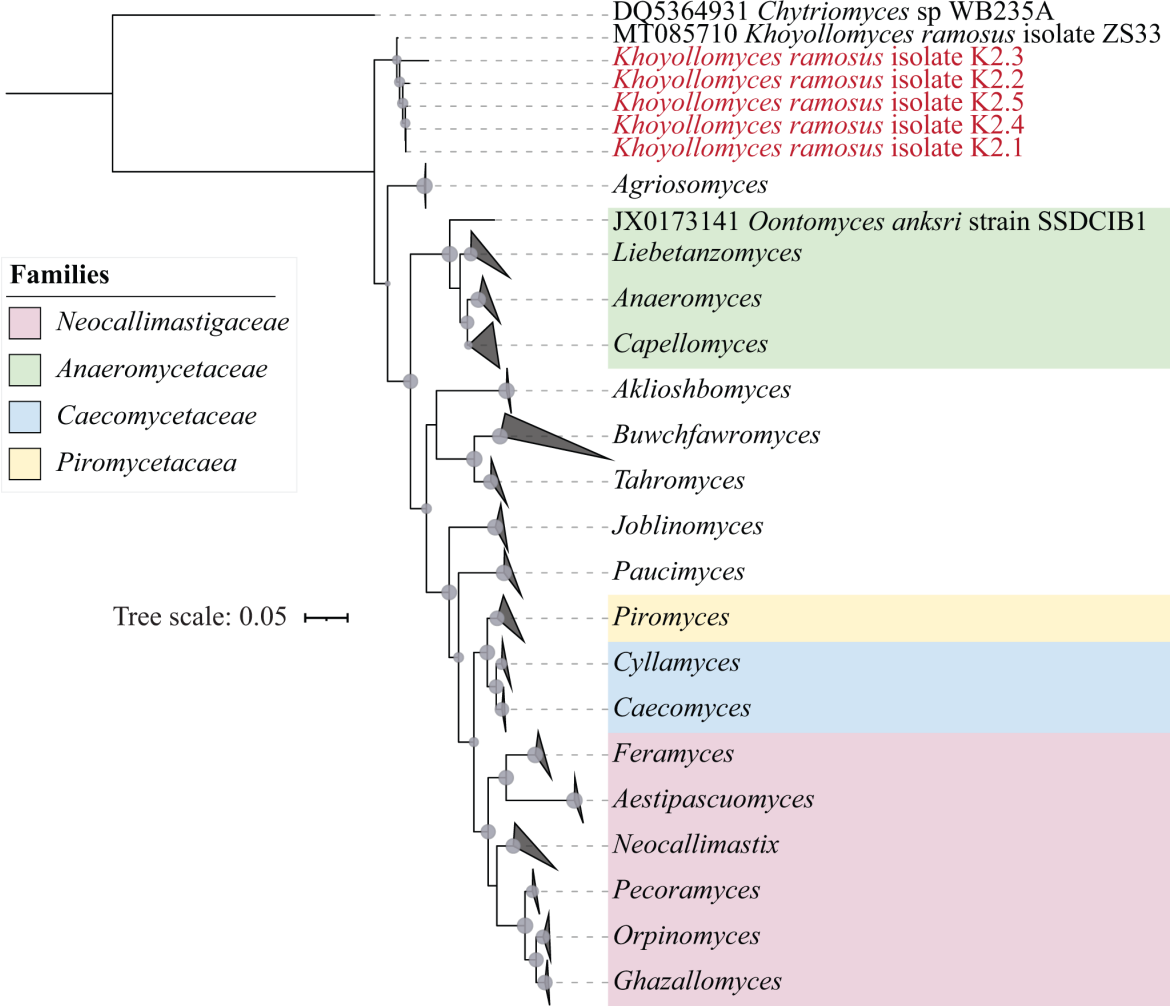
Assessment of the phylogenetic position of the newly obtained isolates from a kangaroo using D1/D2 LSU as a phylogenetic marker. The tree was constructed using the maximum likelihood approach implemented in FastTree. Scale bar indicates the number of substitutions per site. Bootstrap values are shown for nodes with >70% support as gray spheres, where the size of the sphere is proportional to the bootstrap value. The four previously suggested *Neocallimastigomycota* families are color coded as shown in the figure legend. New isolates are identified as *Khoyollomyces ramosus* and are shown in red text.

Attempts were made to obtain AGF isolates from freshly collected marsupial fecal samples. Despite our best efforts, isolation was only successful from 1 red kangaroo sample out of 40 different enrichment attempts (purple text in Table S1). Five isolates were obtained from a single red kangaroo sample (Table S1). The five isolates were identified as *Khoyollomyces ramosus* (Fig. 7), and their D1/D2 LSU markers were 0.95%–3.6% divergent from the *Khoyollomyces ramosus* type strain ZS33 (GenBank accession number MT085710).

## DISCUSSION

In this study, we investigated the AGF community in marsupial hosts. AGF occurrence was identified in 61 of 184 samples. The AGF communities in marsupials were dominated by genera previously identified as predominant members of the placental mammalian gut mycobiome (Fig. 1 and 2) (27). Diversity and community structure patterns were comparable across all marsupial samples (Fig. 3 and 5) regardless of the animal host family, species, gut type, habitat, or nutritional classification. Assembly of AGF communities is predicted to be largely shaped by stochastic (mostly drift) rather than deterministic processes (Fig. 4). Furthermore, marsupial AGF communities were highly similar to those encountered in foregut, but not hindgut, placental herbivores (Fig. 5). Repeated attempts to isolate AGF from marsupials yielded only five closely related isolates that were <3.6% divergent from the type strain of *Khoyollomyces ramosus* previously isolated from a zebra (24) (Fig. 7). These results collectively indicate that the AGF communities in marsupials are neither novel nor unique and show little signs of selection based on ecological and evolutionary factors.

As described above, we hypothesized that, given their unique gastrointestinal tract structures, dietary preferences, and geographic range restriction, marsupials’ AGF communities could exhibit significant differences in diversity and community structure when compared to their placental counterparts. However, our results indicate that marsupial AGF communities were neither novel nor unique, but rather dominated by well-characterized genera and candidate genera previously identified as predominant taxa in the placental gut (27). Furthermore, given the differences in gut type (sacciform and tubiform enlarged foregut, enlarged caecum, enlarged colon, or enlarged caecum and colon) and dietary preferences (browsers, grazers, and mixed feeders) between various marsupial species examined, we expected AGF communities to display clear distinctions based on ecological or host-associated factors. Surprisingly, we found comparative levels of alpha diversity and highly similar community structure patterns encountered across all marsupial samples. Such lack of clear differences in AGF diversity and community structure between marsupials is in stark contrast to the clear host-driven stratification of AGF communities in placental mammals (27).

Our results demonstrate that the AGF community structure in marsupial and foregut mammalian hosts is highly similar. This failure to identify distinct AGF community in marsupials, as well as the failure to identify novel marsupial-specific AGF genera, strongly suggests that marsupial host evolution was not associated with a parallel process of evolution of marsupial-specific AGF lineages. The reason for the failure of AGF to co-evolve with marsupial herbivores, similar to what has been observed in mammalian herbivores (27), is currently unclear. One possible reason is the difference in the digestive tract architecture, specifically the development of a true rumen chamber in foregut placental mammals and the lack of a similar process in marsupials. Another possibility could be the marsupials’ ability to forage on poor nutritional diets with lower proportion of cellulose and arabinoxylan hemicellulose, the preferred substrates for AGF. A third possibility would be the historic paucity and absence of land tortoises in Australia, recently shown as harboring novel ancient genera of AGF that could possibly represent the seed ancestors of AGF in placental mammals (44). However, we could not conclusively rule out the potential occurrence of such a process in extinct marsupials or in hosts not sampled in this study. Furthermore, the lack of clear AGF community structure differences between various marsupial hosts based on ecological and evolutionary factors suggests a passive acquisition from foregut placentals. The reason behind the exclusive acquisition of AGF community in all marsupials, regardless of their gut type, from placental foregut donors remains unclear but could possibly be attributed to the higher number of placental foregut animals (e.g., cattle, goat, and sheep) compared to hindgut fermenters (e.g., horses), hence allowing higher incidences of contact and transmission through fecal exposure.

The important role played by AGF in plant biomass degradation in placental mammals has long been recognized. AGF were shown to initiate plant biomass colonization (45, 46) and produce a wide array of highly efficient lignocellulolytic enzymes (19, 47–55). However, their role and relative contribution to plant biomass degradation in marsupials remain unclear. Quantification of AGF load using qPCR showed significantly lower levels (expressed as ribosomal operon copy number per gram of feces) compared to placental mammals (Fig. 6). Also, PCR amplification failed in 62.5% of the samples examined, and enrichment attempts were only successful in 1 of 18 samples. These low AGF loads, especially when coupled to the observed lack of host-selection patterns (Fig. 5), high level of stochasticity (Fig. 3), and apparent passive acquisition patterns from placental hosts, collectively point to a minor role for AGF in marsupial feed digestion. This could be a reflection of marsupial preference to a wider range of diets, many of which have a lower proportion of cellulose and arabinoxylan hemicellulose, the preferred substrates for AGF (19). Whether AGF abundance in marsupial gut microbiomes and their relative importance in the digestive process dynamically vary in individual subjects based on diet composition (e.g., increasing in kangaroos fed fresh grass diet but decreasing when browsing on shrubs) remains to be seen.

The role and relative contribution of vertical (mother to offspring) versus horizontal (acquisition through direct contact or exposure to fecal matter of other animals) transmission in maintaining communities are largely unknown. The observed low AGF load could potentially hinder effective vertical transmission and render the community more prone to loss under adverse conditions (e.g., scarcity of diet, changes in diet composition, sickness, and dysbiosis). This could necessitate continuous horizontal transmission (through direct animal-to-animal contact or exposure to fecal matter) from other marsupial or placental subjects. Evidence of long-term survivability of AGF in dried feces, possibly through the formation of long-term survival structures (19, 56, 57), has previously been reported, a trait that can facilitate cross-subject horizontal transmission in AGF. The proposed continuous need for horizontal transmission and the proposed minor role for AGF in the marsupial gut could account for our inability to detect AGF occurrence in 123 out of 184 samples examined. On the other hand, the high transmissibility of AGF could also facilitate vertical transmission aided by the close proximity associated with extended nurturing and caring of offspring in marsupials.

The geographic isolation of Australia from Gondwana occurred approximately 100 Mya, resulting in the complete separation of Australia from Antarctica (≈45 Mya) and South America (≈30 Mya) (58). The dominance of marsupials throughout Australia’s natural history post-separation from Gondwana, as well as the lack of native placental mammals in Australia, has been well documented (59). As such, given the central role played by placental mammalian evolution in shaping AGF evolution, maintenance, and dissemination (27); the proposed lack of a parallel process in marsupials; and the proposed role of placental hosts in seeding marsupial gut microbiomes with AGF, timing the acquisition of AGF by marsupial hosts represents an interesting dilemma. The lack of historic interaction between placental and marsupial herbivores in Australia represents a bottleneck hindering AGF acquisition during the early stages of marsupial herbivores’ evolution (66 Mya) (60) and subsequent evolution of the order Diprotodontia (53 Mya) (61, 62), the hindgut family Phalangeridae (mid-Eocene, ~45 Mya) (61, 62), the split between Vombatidae and Phascolarctidae (split early Oligocene, ~30 Mya) (61, 62), and the evolution of the foregut family Macropodidae (mid-Miocene, ~15 to 18 Mya) (61, 62).

The only recorded instances of placental mammals arriving in Australia prior to human colonization are bats, rodents, and dugongs visiting the shores of the continents. The colonization of Australia by Aboriginal Australians (≈50,000 years ago) could represent another opportunity for placental mammalian introduction. Conversely, the colonization by European settlers, commencing in the late 1780s, has certainly led to the introduction of multiple placental mammals, including many herbivores, to Australia. Given that timeline, an earlier AGF seeding of marsupials by placentals prior to human colonization appears unlikely, given the lack of AGF in bats and rodent guts and the extreme transient nature of potential interactions between the herbivorous hindgut fermenting dugong with marsupials. As well, while we reason that Aboriginal Australians’ arrival to Australia has introduced some placental species (e.g., dingo), there is no concrete evidence for the wide-scale introduction of AGF-harboring placental herbivores during this earlier wave of human colonization. Therefore, we raise the intriguing possibility that AGF occurrence in marsupial hosts represents a very recent phenomenon enabled by the large-scale introduction of cattle and other large placental herbivores into Australia, post-European colonization.

In conclusion, our study is the first to provide a detailed analysis of the marsupial AGF community. We provide a thorough analysis of the patterns of occurrence, identity, loads, diversity, and community structure of AGF in marsupial hosts and use these results to provide insights on the possible role of AGF in the marsupial gut microbiome, acquisition and retention patterns of AGF in marsupials, co-evolutionary patterns, or lack thereof, between marsupials and AGF, and potential timing of AGF colonization of the marsupial gut.

## SUPPLEMENTAL MATERIAL

Supplemental material mbio.03370-23-s0001.pdf Supplemental tables and figure.

Table S1 mbio.03370-23-s0002.xlsx Summary of data sets examined in this study.

Table S2 mbio.03370-23-s0003.xlsx Placental hosts utilized for AGF diversity and load comparisons.

Table S4 mbio.03370-23-s0004.xlsx Good's coverage and AGF genus-level community composition.

ASM does not own the copyrights to Supplemental Material that may be linked to, or accessed through, an article. The authors have granted ASM a non-exclusive, world-wide license to publish the Supplemental Material files. Please contact the corresponding author directly for reuse.

## References

[1] Arman SD, Prideaux GJ. 2015. Dietary classification of extant kangaroos and their relatives (Marsupialia: Macropodoidea). Austral Ecol 40:909–922. doi:10.1111/aec.12273

[2] Chong R, Cheng Y, Hogg CJ, Belov K. 2020. Marsupial gut microbiome. Front Microbiol 11:1058. doi:10.3389/fmicb.2020.01058 32547513 PMC7272691

[3] Melzer A, Cristescu R, Ellis W, FitzGibbon S, Manno G. 2014. The habitat and diet of koalas (Phascolarctos cinereus) in Queensland. Aust Mammal 36:189. doi:10.1071/AM13032

[4] Hume ID. 1989. Nutrition of marsupial herbivores. Proc Nutr Soc 48:69–79. doi:10.1079/pns19890011 2660159

[5] Freeman MS. 2018. Marsupial diet, p 1–8. In Vonk J, Shackelford T (ed), Encyclopedia of animal cognition and behavior. Springer International Publishing, Cham.

[6] Dhakal S, Boath JM, Van TTH, Moore RJ, Macreadie IG. 2020. Siccibacter turicensis from kangaroo scats: possible implication in cellulose digestion. Microorganisms 8:635. doi:10.3390/microorganisms8050635 32349400 PMC7284360

[7] Ouwerkerk D, Klieve AV, Forster RJ, Templeton JM, Maguire AJ. 2005. Characterization of culturable anaerobic bacteria from the forestomach of an eastern grey kangaroo, Macropus giganteus. Lett Appl Microbiol 41:327–333. doi:10.1111/j.1472-765X.2005.01774.x 16162139

[8] Singh S, Thavamani P, Megharaj M, Naidu R. 2015. Multifarious activities of cellulose degrading bacteria from koala (Phascolarctos cinereus) faeces. J Anim Sci Technol 57:23. doi:10.1186/s40781-015-0056-2 26290743 PMC4540270

[9] Gulino L-M, Ouwerkerk D, Kang AYH, Maguire AJ, Kienzle M, Klieve AV. 2013. Shedding light on the microbial community of the macropod foregut using 454-amplicon pyrosequencing. PLoS One 8:e61463. doi:10.1371/journal.pone.0061463 23626688 PMC3634081

[10] Barker CJ, Gillett A, Polkinghorne A, Timms P. 2013. Investigation of the koala (Phascolarctos cinereus) hindgut microbiome via 16S pyrosequencing. Vet Microbiol 167:554–564. doi:10.1016/j.vetmic.2013.08.025 24095569

[11] Eisenhofer R, Brice KL, Blyton MD, Bevins SE, Leigh K, Singh BK, Helgen KM, Hough I, Daniels CB, Speight N, Moore BD. 2023. Individuality and stability of the koala (Phascolarctos cinereus) faecal microbiota through time. PeerJ 11:e14598. doi:10.7717/peerj.14598 36710873 PMC9879153

[12] Blyton MDJ, Soo RM, Hugenholtz P, Moore BD. 2022. Characterization of the juvenile koala gut microbiome across wild populations. Environ Microbiol 24:4209–4219. doi:10.1111/1462-2920.15884 35018700

[13] Boath JM, Dakhal S, Van TTH, Moore RJ, Dekiwadia C, Macreadie IG. 2020. Polyphasic characterisation of Cedecea colo sp. nov., a new enteric bacterium isolated from the koala hindgut. Microorganisms 8:309. doi:10.3390/microorganisms8020309 32102268 PMC7074957

[14] Brice KL, Trivedi P, Jeffries TC, Blyton MDJ, Mitchell C, Singh BK, Moore BD. 2019. The koala (Phascolarctos cinereus) faecal microbiome differs with diet in a wild population. PeerJ 7:e6534. doi:10.7717/peerj.6534 30972242 PMC6448554

[15] Pope PB, Denman SE, Jones M, Tringe SG, Barry K, Malfatti SA, McHardy AC, Cheng JF, Hugenholtz P, McSweeney CS, Morrison M. 2010. Adaptation to herbivory by the tammar wallaby includes bacterial and glycoside hydrolase profiles different from other herbivores. Proc Natl Acad Sci U S A 107:14793–14798. doi:10.1073/pnas.1005297107 20668243 PMC2930436

[16] Shiffman ME, Soo RM, Dennis PG, Morrison M, Tyson GW, Hugenholtz P. 2017. Gene and genome-centric analyses of koala and wombat fecal microbiomes point to metabolic specialization for eucalyptus digestion. PeerJ 5:e4075. doi:10.7717/peerj.4075 29177117 PMC5697889

[17] Li Y, Steenwyk JL, Chang Y, Wang Y, James TY, Stajich JE, Spatafora JW, Groenewald M, Dunn CW, Hittinger CT, Shen X-X, Rokas A. 2021. A genome-scale phylogeny of the kingdom fungi. Curr Biol 31:1653–1665. doi:10.1016/j.cub.2021.01.074 33607033 PMC8347878

[18] Orpin CG. 1975. Studies on the rumen flagellate Neocallimastix frontalis. J Gen Microbiol 91:249–262. doi:10.1099/00221287-91-2-249 1462

[19] Gruninger RJ, Puniya AK, Callaghan TM, Edwards JE, Youssef N, Dagar SS, Fliegerova K, Griffith GW, Forster R, Tsang A, McAllister T, Elshahed MS. 2014. Anaerobic fungi (phylum Neocallimastigomycota): advances in understanding their taxonomy, life cycle, ecology, role and biotechnological potential. FEMS Microbiol Ecol 90:1–17. doi:10.1111/1574-6941.12383 25046344

[20] Gordon GL, Phillips MW. 1998. The role of anaerobic gut fungi in ruminants. Nutr Res Rev 11:133–168. doi:10.1079/NRR19980009 19087463

[21] Callaghan TM, Podmirseg SM, Hohlweck D, Edwards JE, Puniya AK, Dagar SS, Griffith GW. 2015. Buwchfawromyces eastonii gen. nov., sp. nov.: a new anaerobic fungus (Neocallimastigomycota) isolated from buffalo faeces. MycoKeys 9:11–28. doi:10.3897/mycokeys.9.9032

[22] Dagar SS, Kumar S, Griffith GW, Edwards JE, Callaghan TM, Singh R, Nagpal AK, Puniya AK. 2015. A new anaerobic fungus (Oontomyces anksri gen. nov., sp. nov.) from the digestive tract of the Indian camel (Camelus dromedarius). Fungal Biol 119:731–737. doi:10.1016/j.funbio.2015.04.005 26228561

[23] Hanafy RA, Dagar SS, Griffith GW, Pratt CJ, Youssef NH, Elshahed MS. 2022. Taxonomy of the anaerobic gut fungi (Neocallimastigomycota): a review of classification criteria and description of current taxa. Int J Syst Evol Microbiol 72. doi:10.1099/ijsem.0.005322 35776761

[24] Hanafy RA, Lanjekar VB, Dhakephalkar PK, Callaghan TM, Dagar SS, Griffith GW, Elshahed MS, Youssef NH. 2020. Seven new Neocallimastigomycota genera from wild, zoo-housed, and domesticated herbivores greatly expand the taxonomic diversity of the phylum. Mycologia 112:1212–1239. doi:10.1080/00275514.2019.1696619 32057282

[25] Bauchop T. 1989. Biology of gut anaerobic fungi. Biosystems 23:53–64. doi:10.1016/0303-2647(89)90008-7 2560409

[26] Liggenstoffer AS, Youssef NH, Couger MB, Elshahed MS. 2010. Phylogenetic diversity and community structure of anaerobic gut fungi (phylum Neocallimastigomycota) in ruminant and non-ruminant herbivores. ISME J 4:1225–1235. doi:10.1038/ismej.2010.49 20410935

[27] Meili CH, Jones AL, Arreola AX, Habel J, Pratt CJ, Hanafy RA, Wang Y, Yassin AS, TagElDein MA, Moon CD, et al.. 2023. Patterns and determinants of the global herbivorous mycobiome. Nat Commun 14:3798. doi:10.1038/s41467-023-39508-z 37365172 PMC10293281

[28] Hanafy RA, Johnson B, Youssef NH, Elshahed MS. 2020. Assessing anaerobic gut fungal diversity in herbivores using D1/D2 large ribosomal subunit sequencing and multi-year isolation. Environ Microbiol 22:3883–3908. doi:10.1111/1462-2920.15164 32656919

[29] Young D, Joshi A, Huang L, Munk B, Wurzbacher C, Youssef NH, Elshahed MS, Moon CD, Ochsenreither K, Griffith GW, Callaghan TM, Sczyrba A, Lebuhn M, Flad V. 2022. Simultaneous metabarcoding and quantification of Neocallimastigomycetes from environmental samples: insights into community composition and novel lineages. Microorganisms 10:1749. doi:10.3390/microorganisms10091749 36144352 PMC9504928

[30] Edwards JE, Hermes GDA, Kittelmann S, Nijsse B, Smidt H. 2019. Assessment of the accuracy of high-throughput sequencing of the ITS1 region of Neocallimastigomycota for community composition analysis. Front Microbiol 10:2370. doi:10.3389/fmicb.2019.02370 31681229 PMC6813465

[31] Schloss PD, Westcott SL, Ryabin T, Hall JR, Hartmann M, Hollister EB, Lesniewski RA, Oakley BB, Parks DH, Robinson CJ, Sahl JW, Stres B, Thallinger GG, Van Horn DJ, Weber CF. 2009. Introducing mothur: open-source, platform-independent, community-supported software for describing and comparing microbial communities. Appl Environ Microbiol 75:7537–7541. doi:10.1128/AEM.01541-09 19801464 PMC2786419

[32] McMurdie PJ, Holmes S. 2013. phyloseq: an R package for reproducible interactive analysis and graphics of microbiome census data. PLoS One 8:e61217. doi:10.1371/journal.pone.0061217 23630581 PMC3632530

[33] Derek HO, Jason CD, Wheeler AP, Dinno A. 2023. FSA: simple fisheries stock assessment methods. Available from: https://CRANR-projectorg/package=FSA

[34] Ning D, Deng Y, Tiedje JM, Zhou J. 2019. A general framework for quantitatively assessing ecological stochasticity. Proc Natl Acad Sci U S A 116:16892–16898. doi:10.1073/pnas.1904623116 31391302 PMC6708315

[35] Stegen JC, Lin X, Fredrickson JK, Konopka AE. 2015. Estimating and mapping ecological processes influencing microbial community assembly. Front Microbiol 6:370. doi:10.3389/fmicb.2015.00370 25983725 PMC4416444

[36] Zhou J, Ning D. 2017. Stochastic community assembly: does it matter in microbial ecology? Microbiol Mol Biol Rev 81:e00002-17. doi:10.1128/MMBR.00002-17 29021219 PMC5706748

[37] Ning D, Yuan M, Wu L, Zhang Y, Guo X, Zhou X, Yang Y, Arkin AP, Firestone MK, Zhou J. 2020. A quantitative framework reveals ecological drivers of grassland microbial community assembly in response to warming. Nat Commun 11:4717. doi:10.1038/s41467-020-18560-z 32948774 PMC7501310

[38] Dixon P. 2003. VEGAN, a package of R functions for community ecology. J Veg Sci 14:927–930. doi:10.1111/j.1654-1103.2003.tb02228.x

[39] Hungate RE. 1969. A roll tube method for cultivation of strict anaerobes. Meth Microbiol 3:117–132. doi:10.1016/S0580-9517(08)70503-8

[40] Elshahed MS, Hanafy RA, Cheng Y, Dagar SS, Edwards JE, Flad V, Fliegerová KO, Griffith GW, Kittelmann S, Lebuhn M, O’Malley MA, Podmirseg SM, Solomon KV, Vinzelj J, Young D, Youssef NH. 2022. Characterization and rank assignment criteria for the anaerobic fungi (Neocallimastigomycota). Int J Syst Evol Microbiol 72. doi:10.1099/ijsem.0.005449 35852502

[41] Katoh K, Standley DM. 2013. MAFFT multiple sequence alignment software version 7: improvements in performance and usability. Mol Biol Evol 30:772–780. doi:10.1093/molbev/mst010 23329690 PMC3603318

[42] Price MN, Dehal PS, Arkin AP. 2010. FastTree 2--approximately maximum-likelihood trees for large alignments. PLoS One 5:e9490. doi:10.1371/journal.pone.0009490 20224823 PMC2835736

[43] Anderson MJ, Walsh DCI. 2013. PERMANOVA, ANOSIM, and the Mantel test in the face of heterogeneous dispersions: what null hypothesis are you testing? Ecol Monographs 83:557–574. doi:10.1890/12-2010.1

[44] Pratt CJ, Meili CH, Jones AL, Jackson DK, England EE, Wang Y, Hartson S, Rogers J, Elshahed MS, Youssef NH. 2023. Anaerobic fungi in the tortoise alimentary tract illuminate early stages of host-fungal symbiosis and Neocallimastigomycota evolution. bioRxiv. doi:10.1101/2023.08.25.554870 PMC10978972 38548766

[45] Orpin CG, Bountiff L. 1978. Zoospore chemotaxis in the rumen phycomycete Neocallimastix frontalis. Microbiology 104:113–122. doi:10.1099/00221287-104-1-113

[46] Edwards JE, Kingston-Smith AH, Jimenez HR, Huws SA, Skøt KP, Griffith GW, McEwan NR, Theodorou MK. 2008. Dynamics of initial colonization of nonconserved perennial ryegrass by anaerobic fungi in the bovine rumen. FEMS Microbiol Ecol 66:537–545. doi:10.1111/j.1574-6941.2008.00563.x 18673390

[47] Cao YC, Yang HJ, Zhang DF. 2013. Enzymatic characteristics of crude feruloyl and acetyl esterases of rumen fungus Neocallimastix sp. YAK11 isolated from yak (Bos grunniens). J Anim Physiol Anim Nutr (Berl) 97:363–373. doi:10.1111/j.1439-0396.2012.01281.x 22369648

[48] Comlekcioglu U, Ozkose E, Yazdic FC, Akyol I, Ekinci MS. 2010. Polysaccharidase and glycosidase production of avicel grown rumen fungus Orpinomyces sp. GMLF5. Acta Biol Hung 61:333–343. doi:10.1556/ABiol.61.2010.3.9 20724279

[49] Lange L, Barrett K, Pilgaard B, Gleason F, Tsang A. 2019. Enzymes of early-diverging, zoosporic fungi. Appl Microbiol Biotechnol 103:6885–6902. doi:10.1007/s00253-019-09983-w 31309267 PMC6690862

[50] Morrison JM, Elshahed MS, Youssef N. 2016. A multifunctional GH39 glycoside hydrolase from the anaerobic gut fungus Orpinomyces sp. strain C1A. PeerJ 4:e2289. doi:10.7717/peerj.2289 27547582 PMC4975031

[51] Morrison JM, Elshahed MS, Youssef NH. 2016. Defined enzyme cocktail from the anaerobic fungus Orpinomyces sp. strain C1A effectively releases sugars from pretreated corn stover and switchgrass. Sci Rep 6:29217. doi:10.1038/srep29217 27381262 PMC4933900

[52] Novotná Z, Procházka J, Simůnek J, Fliegerová K. 2010. Xylanases of anaerobic fungus Anaeromyces mucronatus. Folia Microbiol (Praha) 55:363–367. doi:10.1007/s12223-010-0059-9 20680572

[53] O’Malley MA, Theodorou MK, Kaiser CA. 2012. Evaluating expression and catalytic activity of anaerobic fungal fibrolytic enzymes native to Piromyces sp E2 in Saccharomyces cerevisiae. Environ Prog Sustain Energy 31:37–46. doi:10.1002/ep.10614

[54] Steenbakkers PJM, Harhangi HR, Bosscher MW, van der Hooft MMC, Keltjens JT, van der Drift C, Vogels GD, op den Camp HJM. 2003. β-glucosidase in cellulosome of the anaerobic fungus Piromyces sp. strain E2 is a family 3 glycoside hydrolase. Biochem J 370:963–970. doi:10.1042/BJ20021767 12485115 PMC1223235

[55] Steenbakkers PJM, Irving JA, Harhangi HR, Swinkels WJC, Akhmanova A, Dijkerman R, Jetten MSM, van der Drift C, Whisstock JC, Op den Camp HJM. 2008. A serpin in the cellulosome of the anaerobic fungus Piromyces sp. strain E2. Mycol Res 112:999–1006. doi:10.1016/j.mycres.2008.01.021 18539447

[56] McGranaghan P, Davies JC, Griffith GW, Davies DR, Theodorou MK. 1999. The survival of anaerobic fungi in cattle faeces. FEMS Microbiol Ecol 29:293–300. doi:10.1111/j.1574-6941.1999.tb00620.x

[57] Milne A, Theodorou MK, Jordan MGC, King-Spooner C, Trinci APJ. 1989. Survival of anaerobic fungi in feces, in saliva, and in pure culture. Exp Mycol 13:27–37. doi:10.1016/0147-5975(89)90005-4

[58] van den Ende C, White LT, van Welzen PC. 2017. The existence and break-up of the Antarctic land bridge as indicated by both amphi-Pacific distributions and tectonics. Gondwana Res 44:219–227. doi:10.1016/j.gr.2016.12.006

[59] Woodburne MO, Case JA. 1996. Dispersal, vicariance, and the late cretaceous to early tertiary land mammal biogeography from South America to Australia. J Mamm Evol 3:121–161. doi:10.1007/BF01454359

[60] Amador LI, Giannini NP. 2021. Evolution of diet in extant marsupials: emergent patterns from a broad phylogenetic perspective. Mamm Rev 51:178–192. doi:10.1111/mam.12223

[61] Mitchell KJ, Pratt RC, Watson LN, Gibb GC, Llamas B, Kasper M, Edson J, Hopwood B, Male D, Armstrong KN, Meyer M, Hofreiter M, Austin J, Donnellan SC, Lee MSY, Phillips MJ, Cooper A. 2014. Molecular phylogeny, biogeography, and habitat preference evolution of marsupials. Mol Biol Evol 31:2322–2330. doi:10.1093/molbev/msu176 24881050

[62] Meredith RW, Westerman M, Case JA, Springer MS. 2008. A phylogeny and timescale for marsupial evolution based on sequences for five nuclear genes. J Mamm Evol 15:1–36. doi:10.1007/s10914-007-9062-6

