# Supplementary material for: Anaerobic gut fungal communities in marsupial hosts
Source: mBio. 2024 Jan 23;15(2):e03370-23. doi: 10.1128/mbio.03370-23 (PMC10865811; doi:10.1128/mbio.03370-23)
Supplement: Supplemental material — Supplemental tables and figure. [file mbio.03370-23-s0001.pdf]

Supplementary material for:

Anaerobic gut fungal communities in marsupial hosts

Adrienne L. Jones[1], Carrie J. Pratt[1], Casey H. Meili[1], Rochelle Soo[2], Philip Hugenholtz[2], Mostafa S. Elshahed, and Noha Youssef[1*]

[1]Department of Microbiology and Molecular Genetics, Oklahoma State University, Stillwater, OK, USA.
[2]Australian Center for Ecogenomics, University of Queensland, Brisbane, Australia

Supplementary tables:

Table S1. Summary of datasets examined in this study. Samples are grouped by animal host family, then animal host species. Gut type, country of origin, habitat, and nutritional classification are also shown. The 61 samples that produced amplicons are shown on top followed by the samples where AGF amplification was unsuccessful. The 43 samples that were used for AGF quantification (using qPCR) are shown in red text, and the one sample yielded isolates is shown in purple text.

Table S2. Placental hosts utilized for AGF diversity (both alpha and beta diversity) and load (qPCR quantification) comparisons to the marsupial hosts studied here. The sample name column correlates to samples studied in a previous global herbivorous mycobiome analysis [27].

Table S3. Initial number of reads, number of merged sequences and final number of sequences remaining after quality control for each of the 61 samples studied. Sample names match the names in Table S1.

Sample	Number of reads	Number of merged sequences	Final number of sequences remaining after quality control
Kangaroo_K3_Snyder	76929	76929	148
Kangaroo_Marsup_45	12170	12170	1521
Kangaroo_Marsup_46	12957	12957	1467
Kangaroo_Marsup_52	13112	13112	749
Kangaroo_Marsup_55	54164	54164	25550
Kangaroo_Marsup_60	4182	4182	691
Kangaroo_Marsup_62	7750	7750	1707
Kangaroo_Marsup_79	6404	6404	483
Kangaroo_Marsup_81	5186	5186	153
Kangaroo_Marsup_82	11092	11092	6609
Kangaroo_Marsup_83	10058	10058	5192
Kangaroo_Marsup_84	6223	6223	763
Kangaroo_RF2_Hawk	68686	68686	98
Koala_Marsup_102	8781	8781	3834
Koala_Marsup_103	14080	14080	1159
Koala_Marsup_109	39017	39017	153
Koala_Marsup_111	9795	9795	3311
Koala_Marsup_114	5811	5811	1100
Koala_Marsup_115	8514	8514	3564
Koala_Marsup_119	3731	3731	157
Koala_Marsup_123	56072	56072	150
Koala_Marsup_133	5351	5351	154
Koala_Marsup_141	1747	1747	168
Koala_Marsup_145	6660	6660	199
Koala_Marsup_146	13872	13872	7290
Koala_Marsup_148	10719	10719	330
Koala_Marsup_15	6063	6063	274
Koala_Marsup_197	34706	34706	163

Koala_Marsup_33	21737	21737	5183
Koala_Marsup_39	17771	17771	8465
Koala_Marsup_43	19147	19147	4937
Koala_Marsup_66	10109	10109	1723
Koala_Marsup_68	6314	6314	637
Koala_Marsup_70	2158	2158	252
Koala_Marsup_71	2471	2471	127
Koala_Marsup_73	1883	1883	121
Koala_Marsup_74	25937	25937	12046
Koala_Marsup_76	6188	6188	316
Koala_Marsup_89	4681	4681	756
Koala_Marsup_90	9002	9002	3153
Koala_Marsup_91	6077	6077	1017
Koala_Marsup_93	5773	5773	1329
Koala_Marsup_96	6746	6746	1048
Koala_Marsup_98	6185	6185	364
Pademelon_Marsup_56	5470	5470	2812
Possum_Marsup_154	27310	27310	16657
Wallaby_Marsup_51	10243	10243	1847
Wallaby_Marsup_59	32268	32268	176
Wallaby_Marsup_63	9363	9363	3596
Wallaby_Zoo_395	22520	22520	6637
Wallaby_Zoo_399	19129	19129	10433
Wallaby_Zoo_401	25485	25485	684
Wombat_Marsup_106	8126	8126	2459
Wombat_Marsup_152	930	930	470
Wombat_Marsup_155	8836	8836	4736
Wombat_Marsup_188	18798	18798	123
Wombat_Marsup_191	7556	7556	1668
Wombat_Marsup_26	8064	8064	4295
Wombat_Marsup_35	11205	11205	4784
Wombat_Marsup_36	16071	16071	4539
Wombat_Marsup_57	2770	2770	432

Table S4. Good's coverage and AGF genus-level community composition (shown as percentage abundance) for the datasets studied. Samples are shown in the same order as in Table S1.

Table S5. Results of Kruskal-Wallis comparisons for the effect of host factors on alpha diversity measures between the marsupial sampled tested.

Host factor tested	Shannon			Simpson			InvSimpson		
	Kruskal-Wallis chi-squared	df	p-value	Kruskal-Wallis chi-squared	df	p-value	Kruskal-Wallis chi-squared	df	p-value
Species	7.31	7	0.397	7.44	7	0.384	7.44	7	0.384
Family	3.02	3	0.389	3.136	3	0.371	3.136	3	0.371
Gut type	0.085	1	0.770	0.015	1	0.902	0.015	1	0.902
Habitat	0.831	2	0.660	1.04	2	0.595	1.04	2	0.595
Nutrition	0.308	2	0.857	0.17	2	0.918	0.17	2	0.918

Table S6. Kruskal-Wallis test and Dunn post-hoc test statistics for the comparison of AGF alpha diversity measures (Shannon, Simpson, and inv-Simpson indices) between marsupial and placental mammals. Significant p-values (<0.05) are highlighted in red.

Placental mammal gut type	Placental mammal species	Marsupial mammal species	Shannon (Kruskal-Wallis Chi-squared = 78.52, df = 14, p-value = 5.32E-11)		InvSimpson (Kruskal-Wallis Chi-squared = 74.98, df = 14, p-value = 2.39E-10)		Simpson (Kruskal-Wallis Chi-squared = 74.98, df = 14, p-value = 2.39E-10)	
			Dunn test Z-statistics	Dunn test adjusted p-value (Holm)	Dunn test Z-statistics	Dunn test adjusted p-value (Holm)	Dunn test Z-statistics	Dunn test adjusted p-value (Holm)
Foregut Ruminant	Cow	Common Brushtail Possum	1.349	0.177	1.055	0.291	1.055	0.291
		Common Wombat	-0.064	0.949	-0.621	0.535	-0.621	0.535
		Eastern Grey Kangaroo	0.937	0.349	1.313	0.189	1.313	0.189
		Koala	-0.029	0.977	0.864	0.388	0.864	0.388
		Red Kangaroo	-0.940	0.347	-0.168	0.867	-0.168	0.867
		Red legged Pademelon	1.537	0.124	1.891	0.059	1.891	0.059
		Red necked Wallaby	-0.411	0.681	0.458	0.647	0.458	0.647
		Southern Hairy Nosed Wombat	0.854	0.393	1.100	0.271	1.100	0.271
	Goat	Common Brushtail Possum	0.846	0.398	1.405	0.160	1.405	0.160
		Common Wombat	-1.017	0.309	0.043	0.966	0.043	0.966
		Eastern Grey Kangaroo	-1.890	0.059	-0.650	0.516	-0.650	0.516
		Koala	1.881	0.060	-0.465	0.642	-0.465	0.642
		Red Kangaroo	0.381	0.703	-1.087	0.277	-1.087	0.277
		Red legged Pademelon	2.040	0.041	1.541	0.123	1.541	0.123
		Red necked Wallaby	0.718	0.473	-0.328	0.743	-0.328	0.743
		Southern Hairy Nosed Wombat	1.902	0.057	0.370	0.711	0.370	0.711
	Sheep	Common Brushtail Possum	1.527	0.127	1.734	0.083	1.734	0.083
		Common Wombat	0.273	0.785	0.665	0.506	0.665	0.506
		Eastern Grey Kangaroo	-0.600	0.549	-0.028	0.978	-0.028	0.978
		Koala	0.704	0.481	1.712	0.087	1.712	0.087
		Red Kangaroo	1.407	0.160	1.949	0.051	1.949	0.051
		Red legged Pademelon	-1.359	0.174	-1.212	0.225	-1.212	0.225
		Red necked Wallaby	0.811	0.418	1.065	0.287	1.065	0.287
		Southern Hairy Nosed Wombat	0.484	0.628	-0.314	0.754	-0.314	0.754

Hindgut	Elephant	Common Brushtail Possum	2.564	0.010	2.480	0.013	2.480	0.013
		Common Wombat	2.124	0.034	1.980	0.048	1.980	0.048
		Eastern Grey Kangaroo	1.374	0.169	1.385	0.166	1.385	0.166
		Koala	-3.282	0.001	-3.209	0.001	-3.209	0.001
		Red Kangaroo	-3.435	0.001	-3.255	0.001	-3.255	0.001
		Red legged Pademelon	0.188	0.851	0.330	0.741	0.330	0.741
		Red necked Wallaby	-2.791	0.005	-2.457	0.014	-2.457	0.014
		Southern Hairy Nosed Wombat	-1.618	0.106	-1.770	0.077	-1.770	0.077
	Horse	Common Brushtail Possum	2.880	0.004	2.973	0.003	2.973	0.003
		Common Wombat	2.813	0.005	2.987	0.003	2.987	0.003
		Eastern Grey Kangaroo	1.955	0.051	2.306	0.021	2.306	0.021
		Koala	-5.520	0.000	-6.060	0.000	-6.060	0.000
		Red Kangaroo	-4.835	0.000	-5.072	0.000	-5.072	0.000
		Red legged Pademelon	-0.008	0.994	-0.041	0.967	-0.041	0.967
		Red necked Wallaby	-3.786	0.000	-3.785	0.000	-3.785	0.000
		Southern Hairy Nosed Wombat	-2.313	0.021	1.917	0.055	1.917	0.055
	Rhino	Common Brushtail Possum	2.130	0.033	1.053	0.293	1.053	0.293
		Common Wombat	1.374	0.170	0.564	0.573	0.564	0.573
		Eastern Grey Kangaroo	0.758	0.448	1.498	0.134	1.498	0.134
		Koala	1.805	0.071	1.805	0.071	1.805	0.071
		Red Kangaroo	2.173	0.030	-0.684	0.494	-0.684	0.494
		Red legged Pademelon	-0.419	0.675	1.315	0.188	1.315	0.188
		Red necked Wallaby	1.797	0.072	-0.821	0.412	-0.821	0.412
		Southern Hairy Nosed Wombat	-0.910	0.363	1.315	0.188	1.315	0.188
	Zebra	Common Brushtail Possum	2.501	0.012	2.590	0.010	2.590	0.010
		Common Wombat	1.936	0.053	2.069	0.039	2.069	0.039
		Eastern Grey Kangaroo	1.320	0.187	1.581	0.114	1.581	0.114
		Koala	2.515	0.012	2.783	0.005	2.783	0.005
		Red Kangaroo	2.817	0.005	2.970	0.003	2.970	0.003
		Red legged Pademelon	-0.047	0.962	-0.012	0.991	-0.012	0.991
		Red necked Wallaby	2.404	0.016	2.413	0.016	2.413	0.016
		Southern Hairy Nosed Wombat	1.498	0.134	1.884	0.060	1.884	0.060

Table S7. Kruskal-Wallis test and Dunn post-hoc test statistics for the effect of host gut type/ Infraclass combination on alpha diversity measures between the marsupial sampled tested and their placental counterparts

Group 1	Group 2	Shannon (Kruskal-Wallis Chi-squared = 63.66, df = 3, p-value = 9.7E-14)		Simpson (Kruskal-Wallis Chi-squared = 59.93, df = 3, p-value =6.1E-13)		InvSimpson (Kruskal-Wallis Chi-squared = 59.93, df = 3, p-value =6.08E-13)	
		Dunn test Z-statistics	Dunn test adjusted p-value (Holm)	Dunn test Z-statistics	Dunn test adjusted p-value (Holm)	Dunn test Z-statistics	Dunn test adjusted p-value (Holm)
Foregut Marsupialia	Foregut Placentalia	-0.276	1.000	0.313	1.000	0.313	1.000
Foregut Marsupialia	Hindgut Marsupialia	0.206	0.837	-0.086	0.932	-0.086	0.932
Foregut Placentalia	Hindgut Marsupialia	0.646	1.000	-0.527	1.000	-0.527	1.000
Foregut Marsupialia	Hindgut Placentalia	5.370	3.2E-07	5.416	2.4E-07	5.416	2.4E-07
Foregut Placentalia	Hindgut Placentalia	7.617	1.6E-13	6.970	1.9E-11	6.970	1.9E-11
Hindgut Marsupialia	Hindgut Placentalia	6.267	1.9E-09	6.663	1.3E-10	6.663	1.3E-10

Table S8. Results of Wilcoxon test results for the effect of host factors on NST values (measured using Bray-Curtis and Jaccard indices) between the marsupial sampled tested.

Host factor	Group1	Group2	NST-group1	NST-group2	Wilcoxon test statistic	p-value	Index used
Family	Macropodidae	Phascolarctidae	0.748	0.725	588237	4.16E-12	Bray-Curtis
	Macropodidae	Vombatidae	0.748	0.757	479427	0.056	
	Phascolarctidae	Vombatidae	0.725	0.757	392183	3.43E-17	
	Macropodidae	Phascolarctidae	0.729	0.700	676887	5.21E-43	Jaccard
	Macropodidae	Vombatidae	0.729	0.730	509653	0.227	
	Phascolarctidae	Vombatidae	0.700	0.730	340738	3.00E-35	
Species	Red_Kangaroo	Eastern_Grey_Kangaroo	0.523	0.899	0	0	Bray-Curtis
	Red_Kangaroo	Koala	0.523	0.724	1103	0	
	Red_Kangaroo	Red-necked_Wallaby	0.523	0.807	8014	0	
	Red_Kangaroo	Southern_Hairy_Nosed_Wombat	0.523	0.704	88591	4.75E-223	
	Red_Kangaroo	Common_Wombat	0.523	0.772	40779	2.02E-277	
	Eastern_Grey_Kangaroo	Koala	0.899	0.724	984893	0	
	Eastern_Grey_Kangaroo	Red-necked_Wallaby	0.899	0.807	733880	1.20E-73	
	Eastern_Grey_Kangaroo	Southern_Hairy_Nosed_Wombat	0.899	0.704	926167	2.95E-239	
	Eastern_Grey_Kangaroo	Common_Wombat	0.899	0.772	893770	1.06E-204	
	Koala	Red-necked_Wallaby	0.724	0.807	170295	4.30E-144	
	Koala	Southern_Hairy_Nosed_Wombat	0.724	0.704	539987	0.001	
	Koala	Common_Wombat	0.724	0.772	230910	8.80E-97	
	Red-necked_Wallaby	Southern_Hairy_Nosed_Wombat	0.807	0.704	784509	6.97E-108	
	Red-necked_Wallaby	Common_Wombat	0.807	0.772	667329	1.02E-38	
	Southern_Hairy_Nosed_Wombat	Common_Wombat	0.704	0.772	316892	5.81E-46	

	Red_Kangaroo	Eastern_Grey_Kangaroo	0.514	0.900	0	0	Jaccard
	Red_Kangaroo	Koala	0.514	0.699	2736	0	
	Red_Kangaroo	Red-necked_Wallaby	0.514	0.810	9914	0	
	Red_Kangaroo	Southern_Hairy_Nosed_Wombat	0.514	0.681	122772	6.60E-188	
	Red_Kangaroo	Common_Wombat	0.514	0.762	47682	3.08E-269	
	Eastern_Grey_Kangaroo	Koala	0.900	0.699	994780	0	
	Eastern_Grey_Kangaroo	Red-necked_Wallaby	0.900	0.810	753657	3.05E-86	
	Eastern_Grey_Kangaroo	Southern_Hairy_Nosed_Wombat	0.900	0.681	931158	7.71E-245	
	Eastern_Grey_Kangaroo	Common_Wombat	0.900	0.762	898528.5	1.18E-209	
	Koala	Red-necked_Wallaby	0.699	0.810	106194	1.45E-204	
	Koala	Southern_Hairy_Nosed_Wombat	0.699	0.681	522524	0.041	
	Koala	Common_Wombat	0.699	0.762	189627	5.09E-128	
	Red-necked_Wallaby	Southern_Hairy_Nosed_Wombat	0.810	0.681	812821	6.01E-130	
	Red-necked_Wallaby	Common_Wombat	0.810	0.762	711657	1.04E-60	
	Southern_Hairy_Nosed_Wombat	Common_Wombat	0.681	0.762	306444	4.06E-51	
Gut type	Foregut	Hindgut	0.739	0.760	351530	6.79E-31	Bray-Curtis
	Foregut	Hindgut	0.725	0.733	420988	4.72E-10	Jaccard
Habitat	Zoo_USA	Sanctuary_Australia	0.699	0.764	399400	3.33E-15	Bray-Curtis
	Zoo_USA	Sanctuary_Australia	0.709	0.734	483525	0.101	Jaccard
Nutritional type	Grazer	Folivore	0.774	0.714	813980	6.86E-131	Bray-Curtis
	Grazer	Mixed_Feeder	0.774	0.785	366192	1.84E-25	
	Folivore	Mixed_Feeder	0.714	0.785	200919	5.65E-119	
	Grazer	Folivore	0.742	0.694	795789	2.03E-116	Jaccard
	Grazer	Mixed_Feeder	0.742	0.781	266694	2.89E-73	
	Folivore	Mixed_Feeder	0.694	0.781	146957	7.15E-165	

Table S9. Results of Kruskal-Wallis comparisons for the effect of host factors on AGF load (measured using qPCR) between the marsupial sampled tested.

Host factor tested	Kruskal-Wallis chi-squared	df	p-value
Species	1.301	4	0.861
Family	0.808	2	0.668
Gut type	0.182	1	0.669

Table S10. Results of Wilcoxon test results for the effect of host infraclass on AGF load (measured using qPCR) between the marsupial sampled tested and their placental counterparts

Host factor	Group1	Group2	Wilcoxon test statistic	p-value
Infraclass	Marsupialia	Placentalia	504	0.0012

Supplementary figures:

Figure S1. (A) Box and whisker plots showing the distribution of Simpson and Inverse Simpson diversity indices for different families, species, gut types, habitats, and nutritional types of the animals studied. Results of Kruskal-Wallis test are in Table S5. (B) Box and whisker plots showing the distribution of Simpson and Inverse Simpson diversity indices for animal species, color coded by their gut type, in comparison to foregut and hindgut placental animal representatives. (C) Results of Dunn post-hoc tests for pairwise infraclass-gut type comparisons. ns: not significant, ****: p-value < 0.0001.

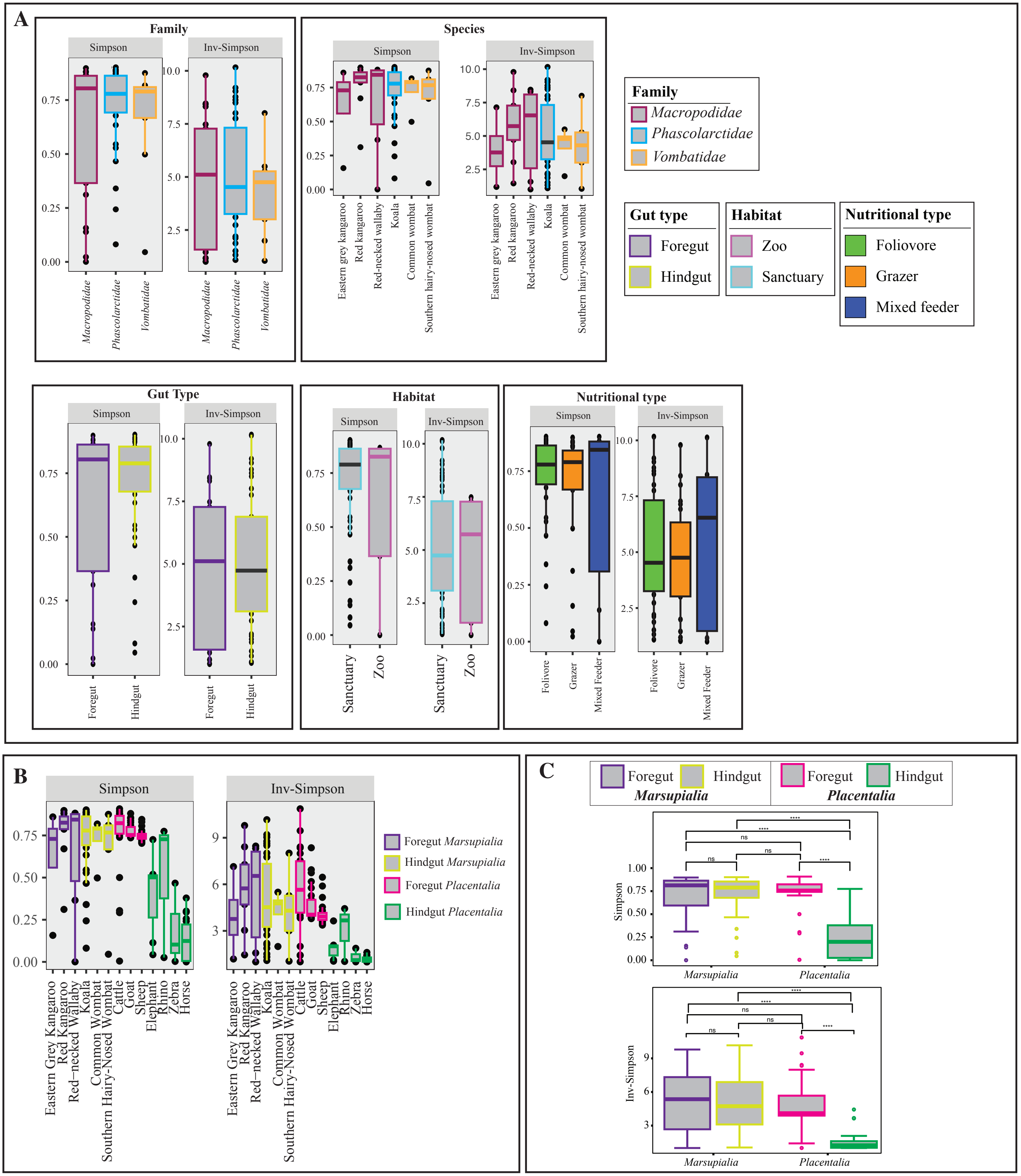
A
Family
Simpson
Inv-Simpson
Macropodidae
Phascolarctidae
Vombatidae
Species
Eastern grey kangaroo
Red kangaroo
Red-necked wallaby
Koala
Common wombat
Southern hairy-nosed wombat
Family
Macropodidae
Phascolarctidae
Vombatidae
Gut type
Foregut
Hindgut
Habitat
Zoo
Sanctuary
Nutritional type
Foliovore
Grazer
Mixed feeder
Gut Type
Habitat
Nutritional type
Folivore
Grazer
Mixed Feeder
B
Simpson
Inv-Simpson
Eastern Grey Kangaroo
Red Kangaroo
Red−necked Wallaby
Koala
Common Wombat
Southern Hairy-Nosed Wombat
Cattle
Goat
Sheep
Elephant
Rhino
Zebra
Horse
Foregut Marsupialia
Hindgut Marsupialia
Foregut Placentalia
Hindgut Placentalia
C
Foregut
Hindgut
Marsupialia
Foregut
Hindgut
Placentalia
Simpson
Inv-Simpson
ns
****
Marsupialia
Placentalia
